# Considerations for Postrelease Environmental Safety Monitoring of Gene Drive-Modified Mosquitoes as a Tool for Malaria Control in Sub-Saharan Africa

**DOI:** 10.4269/ajtmh.26-0049

**Published:** 2026-08-04

**Authors:** Paul K. Abram, Jeremy Bouyer, Brinda Dass, Dorington Ogoyi, Fredros Okumu, Justin Overcash, Stephanie L. James

**Affiliations:** ^1^ Agassiz Research and Development Centre, Agriculture and Agri-Food Canada, Agassiz, British Columbia, Canada;; ^2^ ASTRE, CIRAD, INRAE, University of Montpellier, Montpellier, France;; ^3^ ASTRE, CIRAD, INRAE, University of Montpellier, Plateforme Technologique CYROI, Sainte Clotilde, France;; ^4^ Foundation for the National Institutes of Health, North Bethesda, Maryland, USA;; ^5^ Department of Biology and Life Sciences, Technical University of Kenya, Nairobi, Kenya;; ^6^ School of Biodiversity, One Health, and Veterinary Medicine, University of Glasgow, Glasgow, United Kingdom;; ^7^ Environmental Health and Ecological Sciences Department, Ifakara Health Institute, Ifakara, Tanzania

## Abstract

Progress on malaria elimination has stalled, especially in Africa where approximately 600,000 deaths occurred in 2024. Gene drive-modified mosquito (GDMM) technologies are a potentially transformational new tool to prevent malaria transmission by *Anopheles* mosquitoes. Gene drive-modified mosquito technologies have been successful in contained (caged) testing, and field testing is being planned. Gene drive-modified mosquitoes are designed to be more specific vector control tools than chemical insecticides. However, the potential for spread and persistence of engineered gene drive constructs within interbreeding mosquito populations has raised concerns about possible negative effects on biodiversity. Postrelease safety monitoring of GDMMs will be an important component of risk management, yet scientifically based strategies for evaluating the environmental safety of GDMMs are lacking. Drawing from experience with related technologies in use, we propose a systematic approach to safety monitoring that is risk proportionate, considers ecological priorities and stakeholder concerns, and is practicable to implement in malaria-endemic countries. Suggested methods aim to achieve maximal decision-making value within available resources. Indicators of possible adverse impacts are selected strategically according to ecological analyses using a transparent ranking system. Intensity and coverage of monitoring reflect characteristics of both the GDMMs and the ecological indicator species, with allowance for adjusting the plan in response to initial results, ongoing observations, and regulator feedback. Although developed for early field testing, the framework can also help to guide later phase research and postimplementation environmental monitoring of GDMMs for malaria control in Africa, and it may inform the development of other genetic biological control technologies.

## INTRODUCTION

The WHO proposed a plan to reach 90% reduction in the global malaria burden by 2030.^1^ Despite intensified planning and control efforts initiated under that plan, malaria remains a major health threat for nearly half the world’s population. Although malaria case numbers and deaths declined in the early part of the twenty-first century, this trend has since reversed, leading the WHO to state that global malaria progress has stalled.^2^ There were an estimated 282 million cases and 610,000 deaths in 2024, with 95% of the deaths occurring in the WHO African region^3^ where control and elimination efforts remain particularly challenging. Humanitarian crises, climate change, resource constraints, and biological challenges, such as drug and insecticide resistance, contribute to the faltering progress.^4^ Consequently, there have been consistent calls for development and deployment of innovative malaria control tools to help overcome these challenges.^5^^–^^8^

Human malaria parasites are transmitted through the bite of female *Anopheles* mosquitoes.^3^ One proposed new malaria control approach is the use of genetic technologies to limit parasite transmission^9^ by introducing traits that reduce the ability of *Anopheles* mosquito vectors to reproduce (population suppression) and/or inhibit parasite development in the mosquito (population modification or replacement) (Box 1).^10^ These technologies build on years of experience with other biological control methods.^10^ Classical biological control, which involves the release of known pathogens, parasitoids, or predators to reduce populations of pest organisms such as invasive animal or plant species, has been used in agriculture and conservation for decades.^11^ The sterile insect technique (SIT), a biological and genetic control method, entails ongoing inundative release of large numbers of radiation or chemically sterilized conspecific male insects to decrease the frequency of productive mating by local females, and it has been widely applied to suppress agricultural pests and disease vectors.^12^ Biological control methods aimed at reducing mosquito reproduction or pathogen replication in the vector also include the release of *Wolbachia*-infected conspecific mosquitoes.^13^^–^^15^ Moreover, *Aedes* and *Anopheles* mosquitoes that have been genetically modified to limit reproductive capacity are being released to suppress populations in several locations.^16^

Box 1 **Gene drive terminology^10^**

*Gene drive*—a process that promotes the inheritance of certain genes from generation to generation so that they increase in frequency within a population

*Population modification (also called population replacement)*—a form of biocontrol that reduces the ability of the target vector species to transmit a pathogen

*Population suppression (population reduction)*—a form of biocontrol that reduces the population size of the target vector species

*Self-sustaining gene drive*—a system that promotes persistence of the modification at high frequency within the target vector population

*Low-threshold gene drive*—a self-sustaining drive that is able to spread rapidly from only a small initial release

A variation of such biological and genetic control tools employs engineered gene drive^17^ to help the modification spread and persist in the targeted vector population (Box 1). Gene drive boosts the frequency at which the introduced genes are inherited by progeny from matings between modified and wild-type mosquitoes (much greater than the 50% expected by Mendelian inheritance) so that the modification quickly becomes the predominant genotype in the local population. Different forms of gene drive are being explored, including those that intend to limit the spatial or temporal spread of the modification.^9^^,^^17^ However, self-sustaining low-threshold approaches, in which the transgenic construct is intended to spread and persist indefinitely among interbreeding populations (Box 1), are expected to be the most cost effective, and currently, they are the most advanced. Contained (caged) trials of low-threshold, self-sustaining drives for both population modification and population suppression have demonstrated success in *Anopheles*.^18^^,^^19^ If such successes can be replicated in field trials, gene drive-modified mosquitoes (GDMMs) promise to be a transformative new tool, offering easy to deliver, low-cost, precisely targeted, and durable solutions to some of the most difficult malaria control challenges.^20^^,^^21^ Yet, some of the characteristics that make GDMMs such attractive control tools have raised concerns about potential risks of engineered gene drive organisms for the environment.^22^^–^^25^ These concerns include potential unintended adverse environmental effects deriving, for example, from unexpected mutations outside the gene drive target site (off-target effects), outcrossing and spread of the transgenes beyond the intended target organism, or consequences of reducing the abundance of the target mosquitoes on other organisms (see the section Prerelease risk assessment of GDMMs will inform the scope of postrelease safety monitoring).

The WHO recommends a phased pathway for testing and implementation of genetically modified mosquitoes, including GDMMs, beginning with contained (laboratory and caged) studies and moving to field releases of increasing size and complexity.^9^ This guidance calls for comprehensive risk assessment before each new phase of testing, with monitoring of efficacy as well as safety for health and the environment during each testing phase and after implementation (postmarketing) as a public health tool for malaria control. Planning for initial field testing is underway,^26^^–^^28^ and all testing will require prior regulatory approval.

Approval for field release of GDMMs at any phase of development is expected to require an accompanying plan for postrelease monitoring. Monitoring for efficacy after early releases of low-threshold GDMMs will likely involve assessment of whether the gene drive system behaves as expected in the local mosquito population in terms of increased frequency, spread, and stability (genetic efficacy) as well as assessment of the impact on vectorial capacity of the targeted mosquito species (entomological efficacy). Impact on malaria transmission (epidemiological efficacy) can be assessed in later appropriately scaled trials.^9^^,^^28^ Methods exist for assessment of effects on human health, and they are being applied to other new malaria control tools.^29^ Discussions about the most cost-effective techniques for efficacy monitoring on the spatial and temporal scale required for low-threshold GDMMs are currently ongoing in other venues.^28^ However, methods for monitoring environmental safety have not yet been adequately explored. Current guidance for GDMM development^9^ is vague about decision-making criteria for suitable ecological and environmental indicators and the necessary temporal and spatial scale of monitoring for a technology intended to persist and spread in the environment. Thus, clear, proportionate, and reasonable guidelines are needed to design monitoring programs that balance scientific credibility, stakeholder input, and feasibility.

In June 2024, a group of 44 experts from the fields of medical entomology, biosafety, mathematical modeling, and conservation were convened at an international workshop to initiate a discussion of environmental monitoring requirements for early small-scale field releases of self-sustaining low-threshold GDMMs developed to control and eliminate malaria in Africa.^30^ The workshop was supplemented by a number of related webinars.^31^^–^^33^ Here, a working group composed of workshop participants draws upon those discussions as well as experience with other relevant biological control, vector control, and genetically modified organism (GMO) technologies to propose best-practice considerations for monitoring potential ecological risks posed by GDMMs, with an emphasis on recommendations that will be both informative and feasible in the context of malaria-endemic countries. Although this paper maintains a focus on early field testing of self-sustaining low-threshold GDMMs, several aspects discussed here may also be relevant to the later testing and postimplementation phases and more broadly for other forms of genetic biocontrol.

## THE MONITORING PLAN IS BASED ON THE DEFINITION OF HARM TO ENVIRONMENTAL PROTECTION GOALS

The first step in developing a monitoring plan for environmental safety is to set well-reasoned boundaries on what to monitor. Frameworks have been proposed for designing systems to assess a wide range of environmental conditions.^34^^–^^36^ These generally involve determination of desirable ecosystem conditions or protection goals followed by identification of measurable features (indicators) related to those conditions. The United Nations has identified a number of Sustainable Development Goals, among which Goals 14 and 15 for the protection and sustainable use of aquatic and terrestrial ecosystems are particularly relevant.^37^ Also relevant is the Convention on Biological Diversity (CBD), an international agreement that deals with the conservation and sustainable use of biodiversity and equitable use of genetic resources.^38^ Signatories to the CBD are obligated to develop national biodiversity strategies and action plans to operationalize their environmental protection goals, such as those for Kenya.^39^ National environmental protection laws and regulations articulate the valued aspects of the environment to be protected from harm or misuse.

Risk assessment is a process that translates environmental policy into operational steps by identifying which protection goals are relevant to a particular action, how harm to valued ecosystem conditions might result from that action, the likely extent of such harm, and how any identified risks can be mitigated, supporting decisions on what level of environmental impact would be acceptable and/or manageable. Determining whether harm has resulted from the action after it has been initiated requires a priori agreement upon how the harm from a previously identified pathway will be defined as well as a level of impact that equates to unacceptable or actionable. In turn, this necessitates feasible methods for measuring environmental impact and criteria for establishing a causal linkage between the action and the impact.

The Cartagena Protocol on Biosafety (CP),^40^ which provides a legal framework for assessing and managing potential adverse effects of living modified organisms (equivalent to GMOs) on biodiversity, sets out procedures for risk assessment, risk management, and transboundary movements for signatories. Organisms containing engineered gene drives have been determined to meet the definition of living modified organisms under the CP.^41^ Countries that are signatories to the CP are obligated to apply certain biosafety requirements,^42^ including scientifically sound prerelease risk assessment,^43^ as a mechanism to identify and evaluate potential adverse effects of GMOs on conservation and biodiversity as well as human health.^44^

The CBD makes specific reference to the need for monitoring (Article 7),^45^ mandating parties to, as far as possible and as appropriate, monitor the components of biological diversity important for its conservation and sustainable use; to identify processes and categories of activities that may have significant adverse impacts; and to monitor the effects of such activities through sampling and other techniques. Further clarification is contained in Annex III of the CP,^43^ which provides high-level principles and methods for risk assessment and states that “where there is uncertainty regarding the level of risk, it may be addressed by requesting further information on the specific issues of concern or by implementing appropriate risk management strategies and/or monitoring the living modified organism in the receiving environment.” This guidance will pertain to monitoring of GDMMs in African countries that are parties to the CBD and the CP.

## MONITORING EXPERIENCE WITH RELATED TECHNOLOGIES PROVIDES RELEVANT PRECEDENTS

The basic characteristics of GDMMs overlap considerably with those of various other insect control techniques in terms of the intended entomological effect, spatial reach, temporal persistence, specificity, and use of genetic modifications (Table 1). Each of these pest management tactics is associated with some degree of environmental risk.^54^ That is, none of the generalized characteristics of GDMMs are entirely novel compared with chemical and biological methods that already are in use for controlling insect pests and disease vectors. Thus, understanding accepted regulatory and monitoring practices for related technologies, which are summarized here (see Supplemental File 1 for a more comprehensive review), can help to inform what is reasonable and proportionate for GDMMs.

**Table 1:** Characteristics of low-threshold, gene drive-modified mosquitoes compared with some other vector and pest control modalities (see Supplemental File 1 for details)

Insect Control Modality	Intended Entomological Effect	Spatial Reach	Temporal Persistence	Specificity	Genetic Modification*	Regulatory Requirements for Postimplementation Monitoring of Nontarget Environmental Effects
Low-threshold population suppression gene drives	Suppression of vector populations	Self-dispersing	Indeterminate	Single species or interbreeding species complex	Yes	To be determined.
Low-threshold population modification gene drives	Replacement of vector populations with individuals with reduced vector competence	Self-dispersing	Indeterminate	Single species or interbreeding species complex	Yes	To be determined.
Classical biological control	Suppression of pest populations	Self-dispersing	Indeterminate	Single species or few closely related species	No	Postrelease monitoring generally focuses on efficacy, and long-term monitoring of NTOs has been done very rarely.^†^ Plans for postrelease monitoring on selected nontarget organisms are required as part of permit applications in some jurisdictions but not others.^‡^ Postrelease monitoring is usually not required or enforced in most jurisdictions.^‡^
Genetic modification of crop plants for insect pest control	Mortality of invertebrate herbivores feeding on modified crop plants	Localized	Limited	Members of an insect order feeding on the plant	Yes	Postmarket monitoring generally focuses on efficacy. Requirements for environmental monitoring vary among countries.^§^ Both case-specific and general monitoring are needed in the European Union and some other countries.^§^
SIT	Suppression of pest populations	Self-dispersing	Limited	Single species	Yes but not transgenic	Not required in mosquito SIT programs. Sometimes required in tsetse SIT programs, mainly because it is combined with the use of insecticides.^¶^
Release of *Wolbachia*-infected insects for vector control (males only)	Suppression of pest populations	Localized	Limited	Single species	No	Not required.^‖^
Release of *Wolbachia*-infected insects for disease control (bisexual)	Replacement of vector populations with individuals with reduced vectoring competence	Localized	Indeterminate	Single species	No	Not required.^#^
Broad-spectrum insecticides or biopesticides	Suppression of pest populations	Intended to be localized	Variable	Many species in several different orders	No	Postmarket monitoring generally focuses on efficacy. Environmental safety monitoring is not required to be done by developers but may be performed by government agencies or as research studies.**

NTO = nontarget organism; SIT = sterile insect technique.

* This includes any change in genome resulting from the manufacturing method, which may be introduced by radiation or chemical exposure or by transgenesis.

^†^ See Lynch et al.^46^ and Barratt et al.^47^^,^^48^

^‡^ P. K. Abram, personal observations.

^§^ See Ogoyi et al.^49^

^¶^ See Ciss et al.^50^

^‖^ See Environmental Protection Agency.^51^

^#^ S. O’Neill, personal communication.

** See Derua et al.^52^ and Diarra et al.^53^

Broad-spectrum chemical pesticides used in agriculture and vector control, many of which are toxic to nontarget insects and vertebrates, are widely distributed in the environment.^55^ Regulatory approval relies on prerelease toxicity testing,^56^ whereas postmarket monitoring primarily tracks resistance development (efficacy) and environmental pesticide levels. Monitoring of impacts on nontarget organisms (NTOs) is recommended but generally not required.^57^^,^^58^

Regulatory evaluation of classical biological control agents intended to spread and persist in the environment similarly relies largely on prerelease studies assessing potential effects on likely NTOs.^59^ Because populations of biological control agents can spread and cross national boundaries and can establish in countries that did not participate in their release, some regions have developed harmonized regulatory guidance.^60^^,^^61^ Postrelease monitoring is often recommended but rarely required or enforced.^62^^,^^63^ When conducted, postrelease monitoring generally focuses on the efficacy and spread of the control agent, with occasional retrospective studies examining the effects on NTOs.^64^

Postrelease monitoring in SIT programs likewise focuses primarily on efficacy and operational risk management.^65^ In some cases, environmental monitoring has been undertaken voluntarily or at the request of regulatory authorities, typically assessing changes in the abundance of selected NTOs sharing habitats with the target species or of locally valued species.^50^

A self-sustaining population modification strategy for *Aedes aegypti* involves a natural drive mechanism in which virus-blocking *Wolbachia* bacteria are preferentially transmitted from mothers to offspring, allowing the bacteria to spread and persist in mosquito populations.^66^ Because prerelease risk assessments identified no significant environmental risks,^67^ postrelease environmental safety monitoring has not been required (S. O’Neill, personal communication). Monitoring instead focuses on evaluating the spread of *Wolbachia* in the target species and its effectiveness in reducing arboviral disease transmission.^15^^,^^68^

Because GDMMs are GMOs, monitoring experience with other GMOs is also relevant. Most available guidance concerns genetically modified plants. The European Food Safety Authority (EFSA) describes two approaches to postmarket environmental monitoring (PMEM).^69^ Case-specific monitoring is implemented when risk assessments identify potential risks, important knowledge gaps, or significant uncertainty.^69^^,^^70^ This hypothesis-driven monitoring targets outcomes linked to defined environmental protection goals, and it is conducted for a specified period proportional to the magnitude and severity of potential harm or to verify the effectiveness of risk management measures. In contrast, general surveillance is not hypothesis driven and aims to detect unanticipated adverse effects, often using nontarget species as indicators of ecosystem health. Signals detected through such surveillance require further investigation to determine whether they represent harm and whether they are causally linked to the GMO. This framework has been adopted in several countries outside Europe, including some in Africa.^71^ In practice, however, PMEM of genetically modified crops has focused largely on maintaining efficacy, whereas environmental monitoring methods remain inconsistent and often qualitative, relying on surveys and questionnaires.^72^

Guidance has also been developed for genetically modified animals, including insects.^73^ However, a subsequent review by the EFSA concluded that general surveillance is unlikely to be effective for gene drive-modified insects because detecting ecological change, determining harm, and establishing causality across relevant spatial and temporal scales would be extremely difficult.^74^ Alternative approaches for monitoring unanticipated effects have, therefore, been recommended. Nondriving genetically modified mosquitoes approved for commercial release in Brazil were subject to case-specific, time-limited postrelease monitoring based on issues identified during prerelease risk assessment, with no requirement for general surveillance.^75^

In summary, environmental monitoring is generally not required even when the control agent is expected to persist indefinitely in the environment, cross national boundaries, and/or exhibit toxicity for nontarget species. Safety decisions for non-GMO technologies are largely based on data obtained before field use, and postimplementation monitoring usually emphasizes ongoing efficacy. Although it may be encouraged, postrelease monitoring of effects on biodiversity or ecosystem services is not commonly required for release of living biological control agents, including mosquitoes that are not genetically modified. Genetically modified organisms are considered in the context of risk assessment and risk management as described in the CP in many countries, with expectations for both case-specific monitoring and general surveillance in some. However, postimplementation monitoring also focuses in large part here on efficacy, and application of environmental monitoring requirements has not been uniform.

## PRERELEASE RISK ASSESSMENT OF GDMMS WILL INFORM THE SCOPE OF POSTRELEASE SAFETY MONITORING

As with the technologies described above, prerelease risk assessment of GDMMs is expected to provide an important basis for monitoring decisions. International guidance emphasizes case-by-case risk assessment for GDMMs because of the diversity of types of gene drive modifications, species in which gene drive may be applied, and ecosystems/geographies where the gene drive-modified organism may be used.^9^^,^^76^ Signatories to the CP recently welcomed comprehensive risk assessment guidance developed specifically for living modified organisms containing engineered gene drives, with a focus on GDMMs.^77^ This additional voluntary guidance, which complements CP Annex III, describes a science-based approach following a widely accepted stepwise pathway.^78^ This begins with problem formulation, which entails identification of potential adverse effects (harms) relevant to national legislation and concerns of stakeholders in the release area, followed by analysis of the plausibility of these adverse outcomes occurring (pathways to harm). The next stages of risk assessment involve characterization of the potential routes of exposure and magnitude/consequence of the potential harms that were identified as well as prediction of the overall level of risk. Subsequently, decisions about risk management, including postrelease monitoring, can be made. Postrelease environmental monitoring can play a role in validating assumptions made during risk assessment, resolving or reducing residual uncertainty and stakeholder concerns, evaluating the sufficiency of implemented mitigation measures by comparing predicted risks with real-world outcomes, and revealing unanticipated adverse effects. This enables iterative reassessment and refinement of the environmental risk assessment, adjustment or enhancement of risk mitigation strategies, and implementation of appropriate remedial action as necessary.

**Figure 1.**
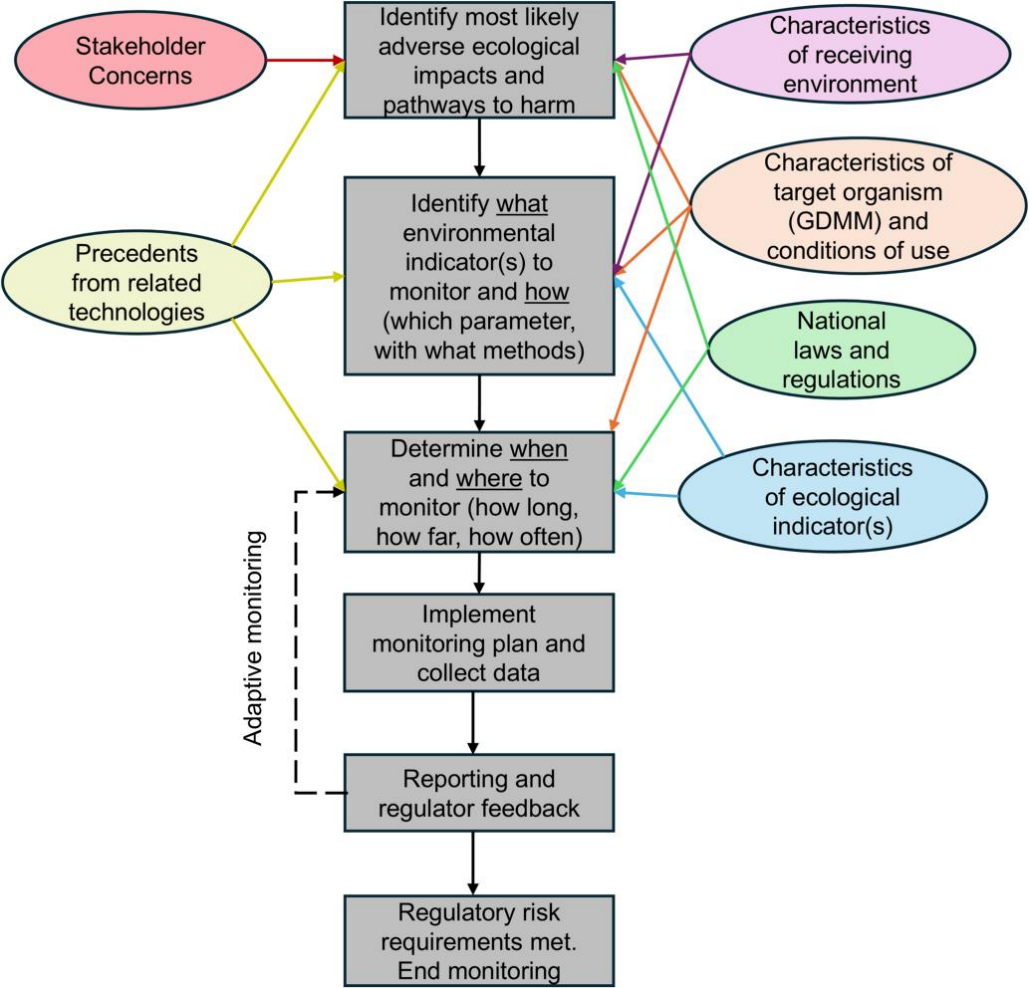
A generic stepwise pathway for designing and implementing the environmental monitoring plan. GDMM = gene drive-modified mosquito.

Figure 1 describes a generic stepwise pathway that can be used to design an environmental monitoring plan applicable to GDMMs as well as other emerging technologies. This pathway begins with case-specific risk assessment to identify and prioritize potential adverse environmental effects likely to result from the release of GDMMs, taking into account the characteristics of the particular GDMMs and receiving environment, the intended conditions of use, and stakeholder concerns. It proceeds to consider what, how, where, how often, and how long to monitor for those residual uncertainties about identified adverse outcomes that remain a concern after risk assessment. These determinations form the basis of the monitoring plan, which must be negotiated and finalized with regulatory authorities. For reasons of practicality, monitoring aspirations should be commensurate with available resources, keeping feasibility and interpretability in mind such that results can inform response decisions. Although the national competent authority^79^ is expected to coordinate decision-making about biosafety monitoring requirements in the parties to the CP, it will be important to consult other ministries, such as those for health, environment, or science and technology, as well as state and local authorities as applicable. As monitoring is implemented and results are reported back to the regulators and involved communities, a need may arise to adapt and modify the existing plan (see the section An adaptive monitoring and reporting framework provides for response to changes). Any subsequent changes to the initial monitoring plan must be approved by the regulators before implementation. Although monitoring requirements during later large-scale testing or after implementation/marketing may differ qualitatively or quantitatively from those during early small-scale field testing, having been informed by the findings of earlier trials, the underlying considerations are likely to remain similar.^9^

Although plans for field testing of GDMMs have not yet been finalized and therefore, risk assessment has not been conducted for any specific product and receiving environment, problem formulation exercises for generic self-sustaining, low-threshold GDMMs have been underway for several years consulting a wide range of stakeholders.^49^^,^^80^^–^^83^ These have identified possible harm to human and animal health, the ecosystem, and biodiversity among the most common general concerns. Reported biodiversity concerns include potential for changes in abundance of mosquito predators because of reduced prey availability, changes in abundance of other mosquito species (e.g., because of competitive release), horizontal gene flow to NTOs, and potential toxic effects on NTOs.^49^ Some have raised the possibility of decreased water quality because of hypothetical effects of GDMM larvae on NTOs in aquatic settings or decreased pollination because of possible changes in nectar feeding as ecosystem services concerns.

Analyses of pathways to harm have been conducted for the most advanced GDMM candidates for population suppression^84^ and population modification^85^ approaches, taking these stakeholder concerns into consideration. Examples of potential pathways to harm are provided in Table 2. Individual steps in each pathway can be interrogated to determine where evidence can be obtained most directly to reduce uncertainty and evaluate the likelihood of harm.

**Table 2:** Previously identified hypothetical pathways of harm to biodiversity or ecosystem services

Mechanism	Net Effect
Population suppression GDMMs*	
Toxicity of transgenic life stages	Predator mortality or reduced reproduction because of consumption of transgenic products; other NTOs suffer damage from exposure to transgenic products (e.g., in aquatic environment)
Increased fitness of transgenics in extreme environments results in geographic range expansion	Increased interspecific competition with NTOs involved in ecosystem services in new range(s) resulting in their displacement or population decline
Reduction in target species creates empty ecological niche	Decreased interspecific competition in empty niche allows for increase in detrimental species (competitive release)
Horizontal gene transfer and functional transgene active/expressed in NTO(s)	Decrease in population of NTO(s) and/or decrease in NTO(s) having ecosystem services function
Increased disease transmission to NTOs by transgenics or by other species whose abundance increases	Increased mortality or decreased population density/fitness of NTOs, including those having ecosystem services function
Increased NTO predation by a predator of target species because of reduction in its chief nutrition source	Decrease in population density of NTOs having ecosystem services function
Increased competition on an NTO providing ecosystem services by a species that has expanded because of suppression of the targeted species	Decrease in population density of NTO having ecosystem services function
**Population modification GDMMs^†^**	
Toxicity of transgenic life stages	Predators die or are less fit because of toxic effects from consumption of transgenic products
Increased fitness of transgenics in extreme environments results in geographic range expansion	Increased interspecific competition with NTOs in new range(s) resulting in displacement or decrease of NTOs having ecosystem services function
Horizontal gene transfer and functional transgene inserted into NTO(s)	Change in population density or behavior of NTOs having ecosystem services function
Decreased consumption of transgenics because of reduced palatability or behavioral changes	Change in population density or behavior of NTOs having ecosystem services function
Increased disease transmission to NTOs by transgenics relative to wild types	Increased mortality or decreased population density/fitness of NTOs having ecosystem services function

GDMM = gene drive-modified mosquito; NTO = nontarget organism.

* Adapted from Connolly et al.^84^

^†^ Adapted from Kormos et al.^85^ and personal communication with Transmission Zero (https://www.transmissionzero.org/).

Data and information used in prerelease risk assessment may be obtained in a weight of evidence manner from the pre-existing literature, expert knowledge and experience, computer simulation modeling, and/or collection of experimental data on the specific or related GMO.^84^ Types of experimental data that can be collected in prerelease contained studies to inform risk assessment include information on toxicity and allergenicity, such as from in vivo (feeding) and in vitro (protein folding database comparison) studies, and changes in developmental or reproductive fitness, temperature sensitivity, insecticide resistance, or vector competence for *Plasmodium* or other mosquito-borne pathogens that might be associated with the gene drive modification in the target species when compared with unmodified target organisms.^9^ In this regard, risks associated with GDMMs should be considered in the context of known risks from other ongoing malaria control methods (Supplemental File 1).

## POSTRELEASE MONITORING CAN ADDRESS UNCERTAINTIES IDENTIFIED IN PRERELEASE RISK ASSESSMENT

Some potential adverse effects may be difficult or impossible to investigate in prerelease studies, often because they involve interactions with other species in complex environments and/or at a scale that cannot be realistically reproduced in contained settings. For these, a lack of prior information could increase uncertainty in initial risk assessment. Postrelease environmental monitoring can provide additional data to better evaluate such uncertainties, which will contribute to better risk management and more informed risk assessment as well as improve the design of future releases. Examples where such uncertainty may remain after prerelease risk assessment based on previous pathways to harm analyses include the possibility that decreased interspecific competition allows for expansion of detrimental mosquito species (population suppression GDMMs in Table 2) and the possibility that horizontal gene transfer (HGT) or some other GDMM-related mechanism has a detrimental effect (e.g., through decreased population density or behavior change) on biodiversity or ecosystem services (population suppression GDMMs and population modification GDMMs in Table 2).

### Horizontal gene transfer.

Horizontal gene transfer (or lateral gene transfer) refers to the stable and heritable acquisition of genetic material not inherited from a parental donor.^86^ The potential for harm related to HGT is often considered in risk assessment for GMOs,^87^ and it has been raised for gene drive-modified insects.^74^^,^^88^ For GDMMs, this risk would require that the introduced genetic material becomes stably integrated into the genome of another organism in a manner that maintains its heritability and functionality in subsequent generations and that this causes harm to the organism or ecosystem in ways relevant to protection goals. Thus, the pertinent questions for environmental safety are as follows. 1) What is the probability that HGT will occur and in a meaningful time frame? 2) What is the probability that an entire functional unit is transferred? 3) What is the probability that it is activated and functional in the new organism? 4) What is the probability that this function will be harmful to the ecosystem through an unacceptable effect on the abundance, distribution, life history/behavior, and/or ecological interactions of the NTO (Table 2) (i.e., the probability of occurrence and severity of the outcome).

Although exchange of genetic material is relatively common in prokaryotes or viruses, it is rare in complex eukaryotes. In those examples where it has been reported, it appears to be vectored by a prokaryote or virus or in extremely rare cases, to be because of incorporation of free DNA from the environment, and it appears to occur only over an evolutionary timescale.^89^^–^^95^ Although GDMMs could use new mechanisms for spread of the construct,^74^^,^^88^ this should not necessarily alter the risk of functional HGT beyond what is currently known from genetically modified plant experience.^86^^,^^87^^,^^96^ Case-by-case assessment as required by the CP and most countries’ laws would analyze the unique combination of specific receiving environments and gene drive constructs from the perspective of likelihood of harm from functional HGT, which can be done using various methods, such as fault tree analysis and comparative genomics.^97^ For example, recent bioinformatics analyses have found no evidence of prior HGT events from mosquitoes to humans or from humans to mosquitoes.^97^

If required, monitoring for the presence of the gene drive construct in other species would be a further step in determining the occurrence of HGT, although additional evidence of construct functionality and associated harm would still be necessary. To examine for the possibility of construct transfer, organisms that interact closely with the target mosquito species could be collected during adult and/or larval sampling of the target mosquito species (see the section Collection and testing methods are available). It will be necessary, however, to carefully exclude species expected to acquire the gene drive construct through vertical transmission (i.e., members of the *Anopheles gambiae* sensu lato species complex)^98^ before testing for its presence in other NTOs.

### Competitive replacement by other mosquito species.

In the case of population suppression approaches that are intended to reduce the abundance of the targeted vector species, concerns have been expressed about competitive replacement either because of an increase in the density of other sympatric mosquito species or because of invasion of new mosquito species. Larval competition has been documented between *Ae. aegypti* and *Aedes albopictus* mosquitoes,^99^ and competitive release, whereby one vector species may be favored by the reduction or elimination of another species, has been observed with these two species.^100^ Interspecific competition among *Anopheles* sibling species also has occasionally been described. For example, the reduction or absence of *An. gambiae* sensu stricto has been reported to favor *Anopheles arabiensis*, even though these two species generally prefer different and only sometimes overlapping habitats and exhibit different host preferences.^101^^,^^102^ Examples also have been documented where *Anopheles funestus* has been locally eliminated by conventional interventions and replaced by less competent malaria vectors.^103^ Competitive release would be most concerning if the new niche was filled by a mosquito species that is a better vector of either malaria or other mosquito-borne diseases, thereby presenting a greater risk to human or animal health than the GDMM-targeted mosquito species. In that case, it would be important to determine that such a change truly represents an increase in the numbers of the undesirable vector rather than an increase in its percentage within a reduced overall mosquito population.

Mosquito species outside the *An. gambiae* s.l. complex, especially known disease vectors, would serve as useful indicators for competitive release. Monitoring will involve looking for long-lasting changes in species diversity or abundance within the local mosquito community that can be causally linked to GDMM release as opposed to other unrelated changes to the environment. Changes in density or diversity of other mosquito species can be examined in the course of adult and/or larval sampling of the target mosquito species conducted for measuring efficacy (see the section Collection and testing methods are available).

### Biodiversity loss and other ecosystem effects.

The concern about loss of biodiversity requires consideration of potential GDMM interactions with NTOs beyond other mosquito species. This may be best addressed by monitoring ecological indicator species with linkages to relevant environmental protection goals following the pathway to harm analysis.^80^^,^^81^ Additional features of an ideal ecological indicator include a high level of direct exposure to the GDMMs, the ability to signal adverse effects rapidly and reliably with sufficient amplitude after exposure to distinguish effects causally linked to the release of GDMMs from other ongoing ecosystem changes, and feasibility (e.g., ease and cost) of sampling and analysis. Where local evidence suggests significant levels of hybridization, the possibility that the gene drive may spread to interbreeding sibling species in the *An. gambiae* s.l. species complex through vertical transmission should be considered in assessing potential NTO interactions.

Methods have previously been proposed for priority selection of NTOs to be considered in prerelease risk assessment of genetically modified plants and insect biological control agents.^48^^,^^104^^–^^106^ Many of these selection criteria overlap with those used to select indicators for monitoring ecosystem health^107^^,^^108^ and could (with relatively little modification) be extended to develop priority rankings of NTOs for postrelease monitoring of GDMMs. Some already have been used to address possible biodiversity impacts of other vector control methods.^50^^,^^109^

Selection of ecological indicators might begin with determining indicator taxa relevant to the identified protection goal and listing species that are known to meet the necessary criteria (e.g., with regard to niche, life history, sample size, and detectable differences in response to natural environmental variation versus changes caused by human activities) and continue by choosing those that are most tractable to being collected and measured. It is important to ensure there are relevant and measurable parameters for each of the identified indicators. One example of a process based on characteristics such as functional importance in the ecosystem and ease of sampling and identification has been used to monitor insects as indicators of habitat and biodiversity conservation.^110^ Another example is the Priority Ranking of Nontarget Invertebrates (PRONTI) system (Box 2), which was originally developed to create prioritized lists of nontarget invertebrate organisms for biosafety testing of transgenic crop plants^104^ and was later extended to developing prerelease NTO test lists for classical biological control agents.^48^ The PRONTI system explicitly lays out the main attributes of organisms that make them good or poor candidates for postrelease monitoring (i.e., how at risk they are, how valued they are, and how tractable they are to monitor) (Table 3), and it encourages users to engage in the focused exercise of evaluating and weighing these attributes in the context of a given proposed monitoring program.

Box 2 **Priority Ranking of Nontarget Invertebrates**

The Priority Ranking of Nontarget Invertebrates (PRONTI) system begins with compiling a list of potential nontarget organisms (NTOs) in the environment, and then, each organism is assigned a PRONTI score, which is calculated using a number of attributes assigned to each NTO:

$$\mathrm{PRONTI}\;\mathrm{score}=\left(\frac{H\times E}{R}\right)\times \left(S+V+T\right),$$

where H is hazard, E is exposure, R is resilience, S is ecological status, V is anthropocentric value, and T is testability (see Table 3 for details on each of these parameters and potential sources of the information).

**Table 3:** Selected criteria and considerations for selecting nontarget organisms to monitor after release of gene drive-modified mosquitoes

Selection Criteria	Examples of Parameters to Consider	Example Sources of Information
**H**azard. To what degree would the stressor pose a hazard to the NTO?	• What the stressor is • Possible direct effects of the stressor on the NTO • Possible indirect effects of the stressor on the NTO	Information in prerelease risk assessment (scientific literature)
**E**xposure. To what degree would the NTO be exposed to the stressor?	• The degree to which the NTO is found in the location and time period where the stressor would be present	Information in prerelease risk assessment (scientific literature)
**R**esilience. How resilient is the NTO to the stressor?	• The degree to which the NTO can avoid the hazard or reduce its exposure (e.g., with dispersal)	Scientific literature
**S**tatus. What is the status of the NTO in the ecosystem?	• The NTO’s biomass • Food web links from the NTO to other organisms in the ecosystem • Other species in competition or even competitive exclusion with the target species • Whether the NTO is known to be a “keystone” species	Scientific literature; prerelease information on regional biodiversity (e.g., from museum collections and existing monitoring networks)
**V**alue. How much do people value the NTO?	• The value of the NTO to human culture • The intrinsic conservation value of the NTO • The economic value of the NTO • The ecosystem services rendered by the NTO • Any other value of the NTO to society (e.g., as a bioindicator species)	Local stakeholder/public/community consultation and surveys in the release area; international/national/regional conservation status list
**T**estability. How feasible is it to monitor the NTO and usefully interpret the resulting data?	• How well the life history and taxonomy of the NTO is known • How easily and inexpensively the NTO can be sampled • How reliably sampling methods reflect true abundance or occurrence of the NTO • How long the generation time of the NTO is (and thus, how likely its populations are to respond on the timescale of the monitoring) • Whether any life stages of the NTO disperse beyond the spatial scale over which the stressor would be expected to act • Whether there are existing monitoring datasets or samples to establish baselines for occurrence or abundance of the NTO • Degree of natural variability in the population abundance/occurrence of the NTO	Consultation with people responsible for existing regional and local biodiversity monitoring programs; published life history studies, monitoring datasets, and sampling studies on the same or similar (closely related or ecologically similar) organisms; published results on previous monitoring of the NTO to measure the environmental impact of other vector control methods

NTO = nontarget organism. Here, the “stressor” refers to any potential result of the presence of the gene drive-modified mosquitoes in the environment (e.g., horizontal or vertical transfer of the genetic construct or reduced biomass of the target mosquito species). The bold letters for each selection criteria refer to the parameters composing the priority ranking calculation (Box 2). Adapted from Todd et al.^104^ and Hilty and Merenlender.^107^

**Figure 2.**
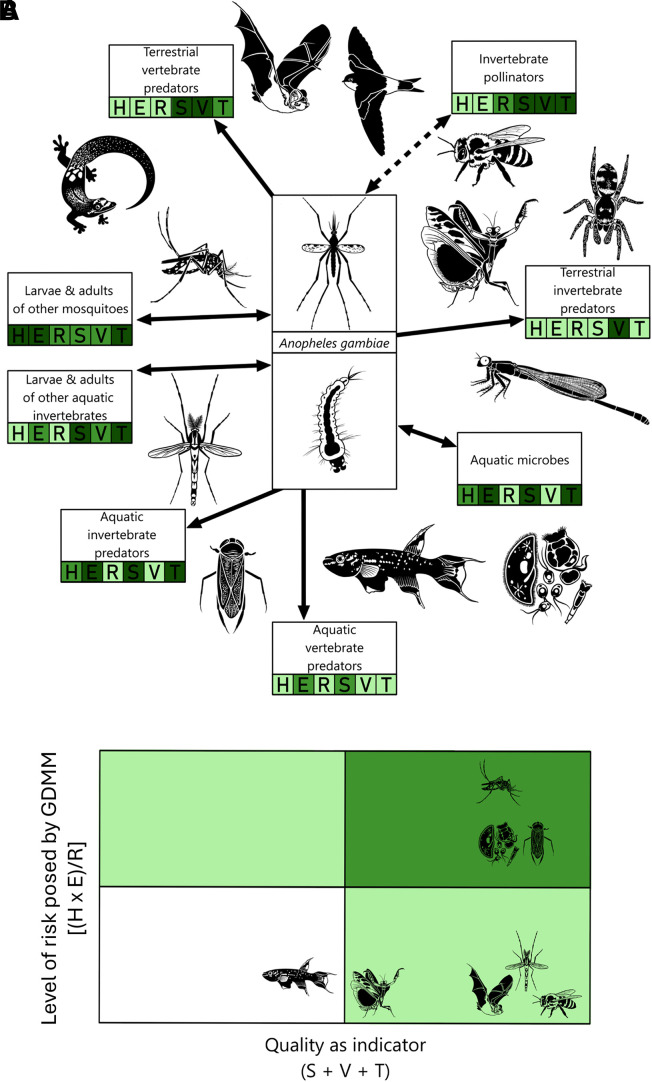
A generic example of potential species interactions with *Anopheles gambiae* sensu stricto gene drive-modified mosquitoes (GDMMs) in sub-Saharan Africa for illustrative purposes. (**A**) A range of types of nontarget organisms (NTOs) with potential relationships to the target mosquito species with hypothetical examples of priority rankings generated by basic calculations. Box 2 has details of the calculation (Supplemental File 2). Darker and lighter shades of green for each letter indicate values of the parameter that would result in higher and lower Priority Ranking of Nontarget Invertebrates scores, respectively. Arrows show the direction(s) of energy flow between pairs of organisms in the food web. Solid and dashed arrows show direct and indirect ecological interactions, respectively. Ecological interactions that could be bidirectional (e.g., pollinators and mosquitoes may share a common generalist predator and thereby, indirectly interact) are indicated with two arrowheads. (**B**) A hypothetical example of how placing NTO characteristics (for a subset of the NTOs in panel **A**) on a four-quadrant plot according to their priority ranking scores could help evaluate the balance between risk posed by the GDMM to each NTO and monitoring feasibility (quality as an indicator) for potential indicator species. Organisms in the upper right quadrant are at relatively high levels of risk from GDMMs and have characteristics that make them tractable indicators relative to the other NTOs considered (in the other three quadrants). E = exposure; H = hazard; R = resilience; S = ecological status; T = testability; V = anthropocentric value.

The advantage of a priority ranking system like PRONTI is that it approaches indicator selection in a systematic way, allowing for input from a range of stakeholders and interest groups, and it can incorporate both empirical and subjective perspectives (Table 3). Literature surveys and baseline studies of the trophic interactions of *An. gambiae* s.s. have begun^32^^,^^33^^,^^111^^,^^112^ and can be used to inform these assessments. Modeling can help to identify relevant indicators by contributing to the characterization of potential ecosystem interactions and genetic, entomological, and ecological effects of GDMMs during risk assessment.^74^ Suggestions for indicators also may come from community engagement activities, National Biodiversity Strategies and Action Plans,^113^ or lists maintained by organizations that monitor endangered species,^114^ which would have a high rating for ecological, cultural, or socioeconomic value in a PRONTI calculation. Figure 2A provides a hypothetical example of potentially relevant species interactions with *An. gambiae* s.s. identified from information reflecting several different sites in sub-Saharan Africa (see Supplemental File 2 for more details).

Systems like PRONTI can also help evaluate the different components of potential ecological indicators, identify trade-offs among these components, and recognize good “consensus candidates” for indicators (Table 3). Calculating PRONTI scores for a given list of NTOs can have several different general outcomes. Some species from the initial NTO list may be very highly valued by people and easy to sample (thus, obtaining high scores for the second half of the equation), but they may be at very low plausible risk from the GDMMs (obtaining low scores for the first half of the equation). Conversely, there may be species that (based on risk assessment) could be at high risk but are very difficult to monitor. The organisms that receive the highest PRONTI scores would be those that are at some risk, are unlikely to be resilient to potential negative impacts, are ecologically important, are valued by people, and would be feasible to monitor. Figure 2B shows for illustrative purposes only how consideration of various characteristics can result in prioritization of NTOs as possible indicators. In this hypothetical exercise, mosquitoes that use similar aquatic breeding sites as *An. gambiae* s.s. ranked highest as potential indicator species in terms of exposure/risk. In developing the monitoring plan for an actual field trial, local experts should be consulted for ranking of NTOs relevant to the field site.

As is the case for any criteria-based down-selection process, it is possible that a priority ranking system like PRONTI may reveal that there are no highly ranked NTOs (upper right quadrant in Figure 2B), and therefore, conducting monitoring that is both implementable and scientifically credible (in terms of its associated risk hypotheses/pathways to harm) might be challenging or arguably, even unwarranted. There could, however, be situations where regulation or stakeholder concern necessitates that some indicator is chosen, even though there is no obviously prioritizable option. In cases where ranking exercises result in relatively low scores for most or all NTOs, visualization of risk and/or indicator quality values can be normalized by dividing them by the highest value among the set of NTOs considered. For the exercise shown in Figure 2B, potential indicators (from Figure 2A) were compared with the most highly ranked NTOs on each of the respective axes, which were other mosquitoes using similar aquatic breeding sites as *An. gambiae* s.s. on the vertical axis and honeybees on the horizontal axis. Thus, even when no NTO ranks highly for both risk and quality, a structured prioritization framework provides transparency and clarity, facilitating consensus-driven decision-making by developers, regulators, and other stakeholders about whether and how to monitor for effects on biodiversity. For example, the Senegalese Ministry of Environment requested environmental safety monitoring of a tsetse elimination program using a combination SIT approach.^115^ Because no pathway to harm was identified and even though they ranked as low risk, agreement was reached on fruit-feeding beetles and butterflies as indicators of ecosystem health based on their high ranking for attributes that make these insect species good indicators for biodiversity and conservation.^109^^,^^110^

Identified concerns about adverse impacts on aquatic ecosystem services largely focus on the possibility of direct or indirect toxicity of GDMM larvae to other aquatic NTOs, allergenicity to humans or animals using the water, or direct or indirect adverse effects on aquatic NTOs arising from changes in the abundance of mosquito larvae.^80^^,^^81^ A risk assessment comparing potential primary and secondary effects on water quality of GDMM larvae with those of different larvicides commonly used to control malaria in sub-Saharan Africa concluded that risk to aquatic ecosystems posed by current larval management methods is likely to be equivalent to or even greater than that associated with GDMMs.^116^ Moreover, prerelease examination for toxic or allergenic characteristics in the gene drive construct would likely be a part of the risk assessment, thereby addressing most questions. If concerns about the potential effects on NTOs remain, these can be addressed by postrelease monitoring of selected indicator species in the larval habitat. Speculation about harm related to decreased pollination of valued plant species also has occasionally arisen in problem formulation exercises.^81^ Although some mosquito species can play a role in the pollination of certain plants, all available evidence indicates that *An. gambiae* s.l. participation in pollen transfer of native, introduced, or beneficial plants is negligible.^117^ Nonetheless, studies directly examining the role of *An. gambiae* in the pollination of African plants are underway to provide confirmatory information.^32^

## COLLECTION AND TESTING METHODS ARE AVAILABLE

Having determined possible ecological indicators to monitor, the next step is deciding upon the collection and testing methods, including the most relevant parameter(s) to be measured and appropriate collection locations as well as suitable sampling and measurement methods. Monitoring plans should be preceded by agreement on standardized protocols for collecting, organizing, accessing, and analyzing the data. Such protocols will necessarily be highly context specific, and thus, they are not discussed in detail here. However, examples of standardized protocols for sampling and measuring a variety of aquatic and terrestrial species as well as data quality metrics are available elsewhere (e.g., National Ecological Observatory Network).^118^ Any subsequent alteration in monitoring methods may interfere with longitudinal data. Thus, measurement methods must be well planned and thoroughly tested in advance before and during collection of baseline data, and therefore, they are not subject to frequent change during field trials.

Mosquito sampling will be required for measuring the genetic and entomological efficacy of GDMMs, but it will also be important for evaluating interactions of GDMMs with other species. Sampling locations may differ for mosquito species that favor different breeding conditions from *An. gambiae* s.s., and this should factor in the choice of indicator species and planning for baseline data collection. Enhanced vector surveillance is one of the pillars of the WHO Global Vector Control Response,^119^ and it includes mosquito sampling that can be used for evaluating GDMM efficacy as well as interactions with other organisms. Many African countries have been using sentinel entomological monitoring as part of their national vector control plans,^120^^–^^122^ and newer platforms are also being established.^123^ Mosquitoes can be captured by standard methods, including trapping of adults and dipping or sweeping for aquatic forms, and analyzed to differentiate species taxonomically or by easily accessible molecular methods, such as polymerase chain reaction (PCR).^124^^,^^125^ Monitoring for the presence of the transgenic construct in the target mosquito species can be done using PCR or possibly, by detection of markers, such as fluorescent proteins encoded in the gene drive construct.^126^ Additional field applicable methods are under consideration (P. Verma, S. Verkuijl, C. K. Yee, F. Randazzo, D. Matoke-Muhia, J. Kayondo, N. Windbichler, M. R. Santos, F. Tripet, J. M. Marshall, unpublished data).

A well-coordinated approach will optimize resource use while maintaining the necessary rigor to evaluate the effects of GDMMs. Synchronizing mosquito and environmental monitoring activities to ensure that no data are wasted can minimize the financial and technical burdens associated with postrelease monitoring. Standard mosquito surveillance traps often collect a wide array of nontarget arthropods referred to as bycatch, which can provide valuable ecological insights beyond their primary vector control purpose.^127^^,^^128^ To enhance the efficiency of monitoring for NTO effects, mosquito collection sites could consider leveraging other organisms caught in traps or aquatic environments. For example, an analysis of a 21-year dataset from the Florida Keys mosquito surveillance program uncovered 98 new *Diptera* distribution records, multiple undescribed species, and clear range-shift signals, illustrating how long-term datasets can track biodiversity change.^127^ Consideration of NTOs most likely to be present in the bycatch from mosquito trapping may factor into the testability component of a priority ranking calculation along with the availability of simple and reliable measurement tools. Although analysis of the bycatch from mosquito collection can be an efficient mechanism for biodiversity monitoring, other types of traps^129^^,^^130^ can also be considered for mass collection of arthropod NTOs if necessary. The utility of retaining a subset of samples for retrospective analysis, if needed, should be considered.

It may be sufficient to identify arthropod NTOs in the bycatch based on morphology, even at higher-order taxonomic levels (e.g., family or order), but newer genomics-based methods hold promise to simplify monitoring, reduce costs, and expand the breadth of detectable environmental effects. Environmental DNA (eDNA) and metabarcoding techniques are already being applied for detection and quantification of mosquito species, and they will be relevant for assessing mosquito population dynamics and competitive release.^131^^–^^134^ Metagenomics and eDNA methods that allow for simultaneous multispecies identification of mixed communities can be applied to NTOs present in the bycatch from adult or larval mosquito collection.^112^^,^^135^^–^^139^ These methods are applicable to other aquatic and terrestrial organisms, and they can be applied across different sample types. Individual organisms may be used for targeted sequencing; pooled specimens may be used for community-level metabarcoding; and environmental matrices, such as air, soil, or water, may be used for passive and noninvasive detection of biodiversity signals.^140^^–^^144^ For example, metagenomics methods might be applied in baseline studies to survey local taxa, from which indicator species could then be prioritized (e.g., using a ranking method) for ongoing targeted sequencing.

DNA sequencing used for taxonomic identification or diagnostic purposes can be performed locally with portable devices^135^^,^^144^^,^^145^; through collaborations with regional laboratories (e.g., Sequel platform^146^); or by sending samples to specialized facilities with high-throughput capabilities tailored to metabarcoding, eDNA, or whole-genome sequencing.^135^^,^^147^ Although these molecular methods are most often used in the detection of the distribution of NTOs, evidence is growing for a correlation between species biomass and eDNA signal, and genomics-based methods may with ongoing technical advances and caveats related to detection bias be adapted to the determination of the relative abundance in the future.^143^^,^^148^^,^^149^ Efforts are also ongoing to develop field-applicable tools for measuring eDNA.^144^

### Collection site location.

Placement of collection sites will be influenced by the scale of testing, habitat suitability for GDMMs, ease of access, agreement of the local community, and ability to obtain any necessary permits. The number of collection sites necessary will be informed by the characteristics of the target and nontarget species of interest as well as the anticipated size and variability of the ecological effect to be measured (see the section Indicator species characteristics influence monitoring intensity). The greater the inherent or environmentally imposed variability of the indicator is, the more difficult it will be to achieve the necessary power to detect a significant effect of GDMMs. Therefore, in choosing study sites, anticipated variability should be minimized to the greatest extent possible as this will influence both the feasibility of the study (e.g., scale, duration, and cost) and the interpretability of results (see the section Interpreting significant effects).

Both longitudinal assessment before and after release and comparative assessment between areas where GDMMs have and have not been released would be useful for the determination of a causal relationship between the release and an observed change in the ecosystem. Release and control (untreated) sites should be chosen to be as similar as possible with respect to mosquito populations and ecological conditions as well as the potential for unrelated ecosystem effects because of influences, such as climate change and socioeconomic development (e.g., urbanization), recognizing, however, that if control areas are too close to release areas, they could lose their value as controls over time because of spread of the gene drive construct.

### Baseline measurements.

Before any release of GDMMs, baseline data should be collected on mosquito populations, nontarget species selected as indicators, and relevant ecosystem processes to establish robust reference points for comparison after the introduction of the gene drive construct. Findings from the prerelease baseline studies will help to target monitoring (e.g., to specific locations, seasons, and niches). Should an adverse effect be observed, determination of causality will be facilitated by information on other potential influences that could be responsible for changes in the indicator(s) being monitored. Thus, developers should also be thinking early about whether and how to track changes unrelated to GDMM release that might affect biodiversity, such as alterations in habitat or climate or the use of other arthropod control methods, during the course of the study.

Baseline data must be collected at both potential control and release sites. Frequency of baseline sampling will need to be appropriate to capture dynamic variations in the densities of target mosquito species as well as the presence and/or abundance of indicator NTOs. Baseline data can provide the basis for determination of what represents a “meaningful” change in abundance of indicator NTOs resulting from GDMM release with respect to both magnitude and duration (see the section Interpreting significant effects). Thus, baseline measurement should continue for a period sufficient to cover likely seasonal or annual fluctuations in local conditions. It is possible, however, that an increase in the number of monitoring sites, representing a range of climatic regions and ecoregions, may partially compensate for a shorter baseline study (i.e., space-for-time substitution).^150^

Standardized protocols and quality control/assurance systems (see the section Collection and testing methods are available) will need to be operational before initiating baseline studies to ensure comparable data across monitoring sites and over time. This will include applications for any permits needed for specimen collection, transport, and analysis. If metagenomics monitoring techniques are to be used, baseline studies will need to include the creation of a bar-code bank associated with morphotype identification.^151^

### Interpreting significant effects.

The challenges of detecting significant trends (increases or decreases) in species occurrence and abundance when monitoring biodiversity are well recognized given the multiple potential sources of variability.^152^^–^^154^ Statistical power analysis^155^ is a common method used to determine whether an observed effect is indeed significant and is an important consideration for designing monitoring plans. Having agreed upon the potential harms and the indicators to be monitored, it will be helpful to decide on a methodology to determine an acceptable range of values for each parameter. Values outside of the range would be interpreted to signal a possible adverse effect requiring further detailed consideration to confirm causality and harm.^156^ In the examples provided above, an adverse effect might be defined as a certain level of increase in the abundance of an undesirable species or a certain decrease in abundance of a valued NTO; this may be evaluated as a difference arising over a specified time period in a longitudinal before and after study, and/or between treated and control areas in a comparative study. Before studies begin, agreement must be reached with regulators about the acceptable range for chosen parameters. Observations from baseline studies can support conclusions about whether a given change falls within the range of normal variability, and therefore, it will be important to consider power analysis in baseline studies as well as during GDMM trials. It will also be necessary to decide collectively what degree of certainty is required to conclude that the unacceptable effect has truly occurred (defined in statistical terms as the percentage confidence level). The monitoring plan then must be designed (or powered) in terms of numbers of sites, frequency of measurements, period of evaluation, baseline data, etc. to enable that level of certainty. These considerations should take the goals of the testing phase into account, especially for early small-scale releases that may be powered to only distinguish very large effects. Examples of power analysis for measurement of trends in abundance of mosquitoes,^157^ other insects,^158^^,^^159^ birds,^160^^,^^161^ and other biodiversity indicators^154^ illustrate how others are approaching monitoring design. For environmental safety monitoring of GDMMs, a further step to determine whether any change that surpasses the agreed-upon level of acceptability is causally linked to GDMM release will be necessary. Observation of an unacceptable adverse effect identified as caused by GDMM release could necessitate changing the monitoring plan or even halting releases entirely (see the section An adaptive monitoring and reporting framework provides for response to changes), which underscores the need to agree on definitions of harm and significance before trial design.

## THE MONITORING PLAN SHOULD DESCRIBE WHEN, WHERE, AND HOW LONG TO MONITOR

For GDMMs, the intensity of monitoring encompasses questions of frequency, spatial scope, and duration. These must be calibrated to balance scientific rigor with feasibility (i.e., comprehensive enough to ensure that ecological and public health risks are minimized while not so demanding as to discourage innovation). As noted above (see the section Monitoring experience with related technologies provides relevant precedents) (Table 1), the characteristics of persistence and spread of an introduced organism are not unique to GDMMs, and experiences with other related technologies can help support decision-making about monitoring intensity. A multifaceted approach that draws on lessons from analogous tools, applies predictive modeling, and engages the relevant stakeholders in respective countries will be best suited to ensure fit-for-purpose monitoring plans that align with the risks, requirements, and opportunities posed by GDMMs.

### Frequency—when to monitor.

The plan should reflect the phased nature of testing and implementation, taking into account the extent of accumulated knowledge and experience with the particular GDMM system. This is consistent with guidance that monitoring should be relevant to uncertainties identified in risk assessment as well as the level of risk posed.^78^ Thus, monitoring may need to be most intensive in early trials. Monitoring may become less frequent once the intervention has become established and risks are better understood. For example, for the tsetse SIT program in Senegal, NTOs were only monitored once a year at the end of the rainy season after demonstrating that both abundance and biodiversity were maximal during this season.^162^ Factors, such as local environmental conditions and ecological complexity, reproductive and dispersal characteristics of the target mosquito species, life history of indicator NTOs (see the section Indicator species characteristics influence monitoring intensity), regulatory requirements, and results of prior risk assessments and postrelease monitoring, can be used to guide the specific monitoring intervals.

For low-threshold GDMMs, modeling suggests that maximum introgression of the transgenic construct and subsequent entomological effect may take place anywhere from several months to a few years after releases begin depending on several conditions, such as release numbers and locations as well as seasonal phenology of the target mosquito population and local geography.^21^^,^^28^^,^^163^ Frequent sampling for ecological effects might best begin when the gene drive construct achieves fixation and entomological efficacy becomes obvious, and it should continue for a period sufficient to reasonably expect the genetic or entomological effect within the target mosquito population to translate to the indicator NTOs (see the section Indicator species characteristics influence monitoring intensity). In later testing phases and implementation, assuming that no adverse effects have been noted previously, frequency may be reduced to be proportionate and responsive to level of risk, focusing on periods of peak mosquito activity (e.g., wet seasons) and/or NTO activity. Over the long term and as the pattern of population dynamics becomes apparent after GDMM release, the monitoring schedules may be reduced even further, and multiyear intervals of sampling could suffice if effects on the target population remain stable and if adverse ecological impacts are not detected (see the section An adaptive monitoring and reporting framework provides for response to changes). Suggested frequencies should be evaluated against spatial scope requirements and data collection methodologies so as to maintain effective risk management without imposing unnecessary burdens on program operations.

### Spatial scope—where to monitor.

For autonomously spreading control tools, the trial plan for monitoring site distribution is expected to rely on spatially distributed sentinel sites and predictive modeling. In pest control research, for instance, dispersal kernels, which estimate the probability of an organism moving a given distance, can guide trap placement and help identify high-risk zones for unintended impacts. Similarly, spatial simulation models have been used to predict the movement of biological control organisms and guide or optimize trap placement.^164^

Guidance for genetically modified plants advises that monitoring be carried out where there is the greatest likelihood of measurable impacts occurring in relation to the level of exposure.^69^ For insects and other animals, this is expected to be the release site.^73^^,^^165^ Despite the potential for expanding spatial distribution, the primary ecological risks of GDMMs are likely to manifest first and most acutely at the release site, where gene drive alleles initially reach high frequencies. Monitoring the spread of the transgenic construct in multiple directions extending out from the release site over a period of approximately 2 years has been proposed for initial field trial design.^28^ During this period, factors impacting the spread of GDMMs should be clarified, and the positioning of monitoring sites can be adjusted accordingly. Predictive modeling software, such as MGSurvE, which incorporates a range of environmental variables (e.g., temperature, humidity, wind patterns, and mosquito life-cycle parameters) can also help anticipate the pathways of GDMM spread.^166^ Factors, such as human-mediated transport (e.g., vehicles or aircraft) or high-altitude wind dispersal of blood-fed and gravid females, could expand the range of possible dispersal.^167^ Monitoring at existing national sentinel vector surveillance sites (see the section Collection and testing methods are available) can be expanded to detect the transgenic construct and ecosystem changes at middle to long distances from release sites. Establishment of additional sentinel mechanisms, including in neighboring countries, may be warranted in regions with documented vector mobility or routine human travel (e.g., transport hubs) to detect long-distance migration events. Ongoing feedback from the sentinel network integrated with predictive models can support iterative refinement of the monitoring plans, balancing resource constraints with the need for robust environmental safeguards.

### Duration—how long to monitor.

Another important planning aspect is the length of the monitoring program. Extended monitoring of biodiversity impact is uncommon for most biological control tools, even when the goal is eradication of the target species and the biological control agent is expected to persist in the environment (Supplemental File 1).

For GDMMs, the duration of monitoring will reflect the remaining degree of risk uncertainty. If no significant adverse effects are observed during the intensive monitoring stage, uncertainty may diminish sufficiently to allow cessation of certain monitoring activities. However, even after the gene drive is established in local mosquito populations and the initial risk profile is better understood, additional monitoring may be requested by regulators or stakeholders to assess for delayed effects on ecosystem processes, such as trophic interactions and food web dynamics. If a change outside the range of normal variability is noted, monitoring should continue (perhaps at enhanced frequency depending on the characteristics of the indicator species) while it is determined whether the change is caused by the GDMMs and represents an adverse effect on the ecosystem. If no significant change is observed, monitoring frequency can be scaled back until appropriate decision-makers agree that it can be safely halted. Stopping conditions should be agreed upon in the monitoring plan.

### Indicator species characteristics influence monitoring intensity.

The intensity of monitoring for effects on biodiversity must take into account both GDMM characteristics of spread and persistence as well as the variability, life cycle, and dispersal characteristics of the selected indicator species. Detecting meaningful adverse effects can require observing multiple generations of NTOs. For example, with suppression-based gene drives, ecological feedback loops, such as predator–prey shifts, could take several generations to become apparent.

**Figure 3.**
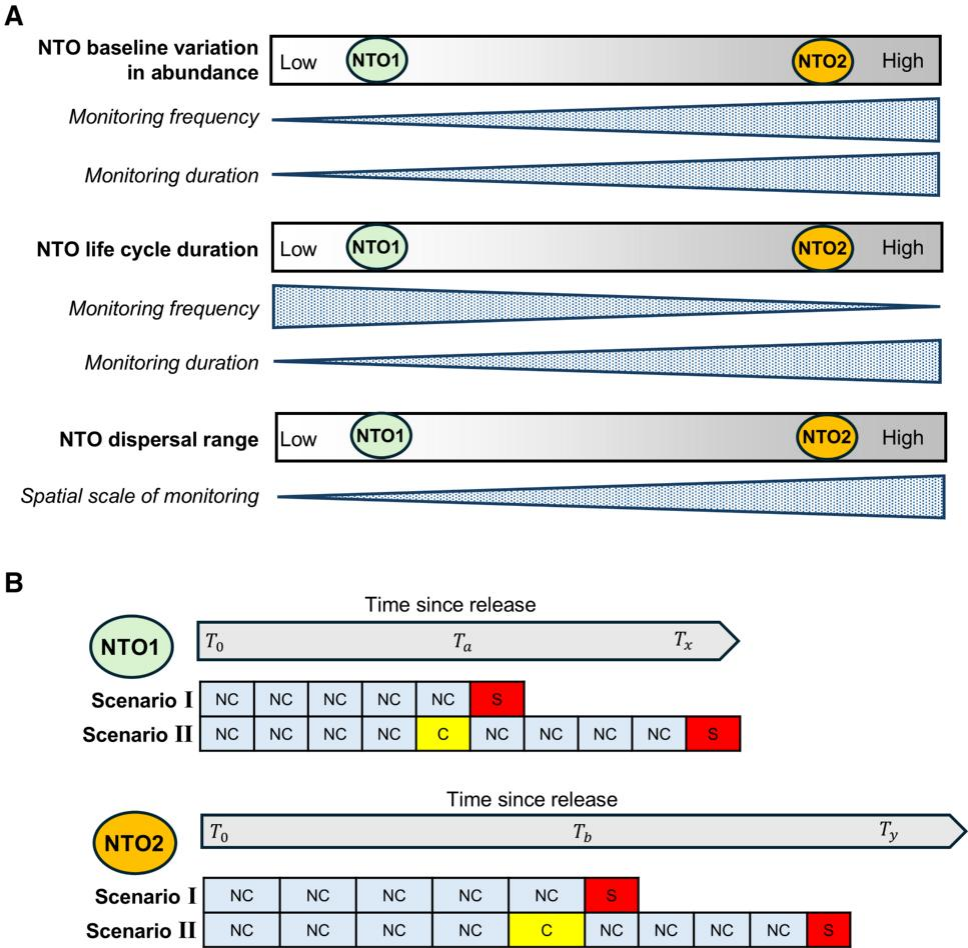
A generalized framework for determining appropriate monitoring intensity and reporting plans for indicator nontarget organisms (NTOs). Consider two hypothetical NTOs (NTO1 in green and NTO2 in orange), each with their individual characteristics. (**A**) The characteristics of each NTO (population variation, life-cycle duration, and dispersal range) will influence the frequency, duration, and spatial scale of monitoring (the continuum is indicated by stippled blue triangles below each characteristic, with wider areas indicating more intense monitoring). The position of each NTO oval on the shaded gray scale indicates where its characteristic is situated and the corresponding appropriate monitoring intensity. (**B**) Example of how NTO characteristics impact overall monitoring duration and reporting needs in an adaptive monitoring scheme. As shown, NTO1 is initially monitored more frequently over a shorter initial duration *T_a_* after release begins at *T*_0_ than NTO2 (monitored until *T_b_* initially) as per their characteristics. Boxes situated below the timescale from the start of release at *T*_0_ represent periods for two hypothetical scenarios. In Scenario I, no significant change is observed in NTO abundance relative to baseline (NC; blue squares), and monitoring stops (S; red squares) after the initial monitoring period. In Scenario II, a change of concern is noted (C; yellow squares), and monitoring is continued at a higher frequency until *T_x_* (NTO1) or *T_y_* (NTO2), where NTO abundance returns to baseline levels for several reporting periods after the change.

Whereas an NTO with a short life cycle might need to be monitored at high frequency for only a short time, an NTO with a long life cycle might reasonably be monitored at low frequency for a longer duration (Figure 3A). Similarly, a relatively sedentary NTO with low dispersal ability could be monitored on a more restricted spatial scale, whereas one with high dispersal ability would need to be monitored on a larger spatial scale reflecting its mobility. An NTO with high baseline variability in population size would need to be monitored for a long duration and sufficient power to enable detection of meaningful change, whereas an NTO with a relatively stable baseline population size could be monitored for a shorter period or less frequently. In addition to baseline observations, the monitoring plan can be informed by life history tables (i.e., data on survival, fecundity, and age structure of the NTO) as well as population modeling approaches that estimate how long GDMMs will retain efficacy.^21^^,^^163^ Overall, NTO species with short life cycles, low dispersal, and low-magnitude natural fluctuations in abundance are likely to be the best ecological indicators.^105^^,^^110^

## AN ADAPTIVE MONITORING AND REPORTING FRAMEWORK PROVIDES FOR RESPONSE TO CHANGES

Monitoring strategies must include reporting procedures and criteria for results-based decisions—such as modifying or stopping monitoring—that are agreed upon with the appropriate regulatory authority. Reporting requirements under national GMO legislation may be applicable. National laws determine responsibility for efficacy and safety monitoring, which typically rests with the applicant (the developer or implementer seeking release approval), although regulatory authorities may retain the right to verify compliance with approved monitoring plans. Monitoring quality may be assessed through data quality control measures, such as periodic auditing. Independent safety oversight during GDMM testing through institutional or national biosafety committees or a project-specific review board can further strengthen confidence in monitoring plans.^9^ Sharing monitoring results will form part of the applicant’s ongoing community engagement responsibilities.

Monitoring plans should specify timelines for reporting results and comply with legal requirements for reporting adverse events. Because reporting timelines may depend on event severity, prior agreement with decision-makers is essential on which outcomes require immediate reporting or remedial action. Applicants will likely be required to include an emergency response plan in regulatory submissions for GDMM releases subject to approval by relevant authorities (consideration of specific mitigation strategies is beyond the scope of this framework). Continued monitoring would then assess the effectiveness of any response. Regulatory authorities may modify monitoring requirements or subsequent release phases based on monitoring results. Applicants also may request adjustments to monitoring plans if new information arises, but regulatory approval must be obtained before implementing changes to avoid breaching permit conditions.

Adaptive monitoring, an iterative approach widely used in conservation and natural resource management,^168^^,^^169^ provides a framework for responding to new ecological information. This tracks ecosystem indicators, evaluates whether objectives are being met, and adjusts monitoring intensity accordingly. As illustrated in Figure 1, monitoring and reporting frequency may increase if unexpected ecological changes occur and decrease or cease if no adverse effects are detected over time (Figure 3B). Adaptive flexibility may also include reducing the number of NTO species monitored during long-term follow-up or adjusting sampling locations according to patterns of GDMM spread. Such an approach allows monitoring to remain responsive to field conditions while improving resource efficiency without compromising oversight. Adaptive monitoring could be particularly important during late-phase testing and postimplementation monitoring when objectives and methods must remain informative yet feasible at larger spatial scales. Provision for incorporating adaptive monitoring provisions into release agreements should, therefore, be negotiated with regulators.

## CHALLENGES AND OPPORTUNITIES EXIST FOR DEVELOPING AN EFFECTIVE MONITORING PLAN

Although experience with other more familiar and widely accepted technologies that share important characteristics with GDMMs provides instructive precedents, challenges can be expected to arise in the development of an effective monitoring plan for GDMMs. Fortunately, opportunities exist for addressing these challenges (Table 4). The success of GDMMs will largely depend upon codevelopment among the scientific establishment, regulatory authorities, malaria control programs, and affected communities in malaria-endemic countries from the start to ensure that the technology is responsive to local values, enhances sustainability, and fosters coresponsibility for monitoring and management.^9^

**Table 4:** Key challenges in environmental monitoring of gene drive-modified mosquitoes and recommended mitigation strategies

Potential Challenges	Recommendations
Difficulties in delineating adequate monitoring area sizes and detecting low-prevalence gene drive elements ○ Gene drives can disperse widely and rapidly, requiring broad geographic coverage ○ Low-density populations at leading edges may remain undetected	Focus intensive environmental monitoring close to the release point where the impact should be maximal Engage community networks (e.g., local volunteers, citizen scientists, and community health workers) to expand sampling coverage into remote or hard-to-reach areas Use rapid data analysis and continuous surveillance to detect unintended spread Use adaptive sampling methods as needed to capture low-density populations at dispersal fronts
Limited baseline data on biodiversity and ecological relationships ○ Ecological roles of *Anopheles* mosquitoes in local food webs are often incompletely understood ○ Complex interactions with NTOs	Integrate environmental sampling into entomological surveillance, preserving nonmosquito bycatch to assess densities and variations of predators/arthropods Begin collecting baseline data well before gene drive deployment for robust comparisons Leverage existing biodiversity monitoring networks to maximize resource use and benefit from established workflows Conduct research on ecological relationships involving *Anopheles* mosquitoes
Unforeseen consequences that can be difficult to plan for ○ Effects that cannot be fully predicted, including emergent ecological or evolutionary outcomes ○ No direct precedents for gene drive mosquito interventions	Monitor control sites where GDMMs are not released to detect unrelated ecological changes Adopt adaptive monitoring frameworks that allow for rapid updates to protocols as new data become available Incorporate iterative risk assessments and feedback loops to regularly refine objectives and methodologies
High costs associated with long-term, extensive data collection ○ Resource constraints in many malaria-endemic regions ○ Need for sustained funding to maintain infrastructure and trained personnel	Use simplified scalable techniques (e.g., eDNA detection and DNA metabarcoding) to increase sampling efficiency Integrate GDMM monitoring into malaria vector surveillance programs to share resources Engage community networks to expand coverage and reduce labor costs Transition to passive long-term monitoring with trained community health workers Establish sustainable funding and multiyear commitments among stakeholders Include monitoring in regulatory requirements, and maintain infrastructure for extended data collection and analysis Reduce monitoring frequency and scope with time if no adverse effects are observed with early releases
Difficulty specifying or achieving statistical power within resource constraints ○ Limited sampling intensity and coverage may reduce the ability to detect significant effects	Engage community networks to broaden sampling scope and frequency Use passive, long-term monitoring strategies (e.g., data collection by community health workers) to increase sampling intervals without dramatically raising costs
Difficulties in detecting rare events or effects ○ Rare but potentially high-impact outcomes (e.g., unintended spread and resistance development) ○ May require specialized methods to identify low-frequency occurrences	Incorporate highly sensitive methods, such as targeted PCR or eDNA sampling Implement broader geographic surveillance to capture potential spread or mutations in distant areas Leverage new statistical approaches and adaptive sampling designs that enhance the ability to detect low-frequency events Regularly review and recalibrate sampling plans based on new data
Lack of established biosafety frameworks in some countries	Provide information on GDMM technologies and support for countries to develop regulatory mechanisms Foster regional collaboration mechanisms to manage issues related to transboundary movement of GDMMs

eDNA = environmental DNA; GDMM = gene drive-modified mosquito; NTO = nontarget organism; PCR = polymerase chain reaction.

A prerequisite for determining where to monitor for environmental safety is knowing the location of the GDMMs. One technical challenge is related to tracking the spread of the gene drive construct, which involves delineating adequate monitoring areas and detecting low-density populations at the leading edges of spread. Because low-threshold GDMMs are designed to spread rapidly, monitoring may need to cover large and perhaps remote regions, and swift data analysis will be needed to detect any unintended spread or unexpected outcomes. This component of environmental monitoring overlaps significantly with efficacy monitoring, the initial phase of which tracks the spread of the gene drive construct within the target mosquito population.^28^ In both cases, monitoring can make use of the widely available mosquito sampling and detection methods described above. With adequate preparation during baseline studies, new molecular methods, such as eDNA and metabarcoding, which are gaining wider acceptance for biodiversity monitoring, may be well suited to detecting the distribution (presence or absence) of NTOs of interest.^149^^,^^170^^–^^172^

Some regulatory authorities may expect a resourcing mechanism to be described in the monitoring plan. Capacity to respond to postrelease commitments has been cited as an ethical obligation of GDMM testing.^9^ To ensure that monitoring aspirations are balanced with financial resources, the utility of new high-throughput and ideally, field-adapted methods that make it simpler to collect and analyze large numbers of samples should be explored in early studies. Any long-term monitoring considered necessary by regulatory authorities or the public may best be accomplished through integration into existing malaria control or biodiversity programs, and these partnerships should be pursued as early as possible.

National or regional programs wishing to establish monitoring systems to detect unintended consequences for effective mitigation will need to ensure that these systems are adaptable and responsive to new information and emerging risks. These challenges may require trained personnel; acquisition and maintenance of specialized equipment and supplies; and a mechanism, such as a relational database, to store data on the location of GDMMs, information on NTOs, and other environmental observations. Another infrastructure challenge relates to data collection across diverse ecological settings in Africa. Reaching remote areas for environmental monitoring can be difficult because of poor road infrastructure and limited transportation options, and even when these concerns are addressed, samples may have to be shipped to centralized locations for storage, analysis, and data processing. Monitoring plans must find ways to collect the necessary data under resource constraints that exist in most malaria-endemic countries: for example, by incorporating environmental monitoring at established sentinel vector surveillance sites.^173^ Development of field-applicable analytical methods would reduce dependence on sample shipment. It may also be possible to take advantage of ongoing innovations and the increasing availability of internet connectivity and smartphones in Africa. Community-sourced, smartphone-based data collection is already proving useful for monitoring certain types of NTOs.^174^^–^^176^ Existing networks, such as national disease control programs and community health workers who have prior exposure to training and data-sharing requirements, are a resource for widespread and long-term sample collection. Cooperation with organizations already active in conservation and biodiversity monitoring within Africa should also be explored. Although baseline biodiversity data in many African countries are still lacking, efforts there are ramping up.^177^

Although several African countries have established national biosafety laws, others potentially interested in GDMMs have yet to create national frameworks. It will be beneficial to provide early opportunities for regulatory authorities and institutional oversight committees to become familiar with the concept of GDMMs and to consider how GDMMs relate to their laws, regulations, and health and environmental priorities before confronting the need to review an application for release. Regional coordination programs could streamline regulatory requirements and unify transport, release, and subsequent monitoring and reporting processes. Existing multilateral cooperation agreements provide examples for this type of regulatory coordination.^42^^,^^60^^,^^61^^,^^178^^,^^179^

Perhaps the greatest challenge of all is that GDMM testing and implementation will occur in the midst of unrelated environmental changes. Africa is experiencing rapid environmental changes because of increasing urbanization and global warming,^180^^,^^181^ which will make it difficult to attribute any observed adverse event solely or principally to the effects of GDMMs. This challenge is not unique to GDMMs and can be mitigated in part by selection of comparison (control) sites with similar ecological and socioeconomic characteristics where biodiversity is monitored in the absence of GDMM releases.

## DISCUSSION: A GENERIC MONITORING FRAMEWORK CAN BE DERIVED FROM PRIOR EXPERIENCE

Although engineered gene drive technologies have sometimes been represented as novel and exceptional because the genetic modifications are purposely intended to spread and persist in target populations,^182^^,^^183^ the scientific community has had decades of experience studying natural drives.^184^^,^^185^ It has been over a decade since the discovery of the clustered regularly interspaced short palindromic repeats (CRISPR)-based engineered gene drive,^186^ and CRISPR-based gene therapy has already been approved for use in humans.^187^ Insights gained during this period support a more nuanced perspective on the characteristics of potential gene drive applications, such as GDMMs; their place in the continuum of genetic biological control technologies^10^; and the technical, regulatory, and ethical requirements for their further development and implementation.^9^^,^^182^

### GDMMs represent a new combination of familiar characteristics.

In considering the need for postrelease environmental monitoring of GDMMs, it is important to reiterate that none of the generalized characteristics of GDMMs are entirely absent from other currently used pest control techniques (Table 1; Supplemental File 1). For example, GDMMs are not the only pest control technology that is self-propagating and irreversible as these are necessary features of all traditional biological control agents. Whereas classical biological control depends on the introduction of entirely new species into a region, gene drive technologies rely on the modification of species that are already present in the ecosystem. In keeping with a comparative risk-based approach, it is informative to examine the relative risks posed by different vector control tools.^116^ Given that gene drive constructs are spread through mating, GDMMs are anticipated to exhibit greater specificity of action than most existing pest control techniques. Notably, the most widely implemented mosquito control technology (and the most likely alternative to GDMMs for malaria control) is the use of pyrethroids, which are broad-spectrum insecticides known to be toxic not only to pest insects but also, to other potentially beneficial and valued species and upon high-level exposure, to humans.^188^^,^^189^ Moreover, although banned in many countries, dichlorodiphenyltrichloroethane (DDT) is still allowed for use against malaria in some developing countries, even though it is known to exhibit toxic effects in vertebrates and long-term persistence in the environment.^190^

Postrelease monitoring of these other common pest control methods typically emphasizes efficacy evaluation. Monitoring of postrelease effects on NTOs and other ecosystem effects for common pest control technologies is encouraged, but it is usually voluntary and only rarely mandated. The expectations for postrelease environmental safety monitoring of GDMMs apparently relate largely to lingering concerns about GMOs and related requirements of the CP. Notably, based on risk assessment, no postrelease environmental monitoring has been required of a self-sustaining natural drive mechanism based on the introduction of a bacterial symbiont in *Aedes* mosquitoes, which has now been conducted in some 15 countries. Reasonable and proportionate monitoring goals for GDMMs should be set with these precedents in mind.

### Needs and feasibility can be balanced through a “Goldilocks” approach.

Guidance on genetically modified mosquitoes issued by the WHO and the CBD calls for assessment of the risk to the environment before release as well as postrelease monitoring for efficacy and safety for health and the environment. As discussed above, environmental monitoring has often been divided into two categories: general and case specific (or hypothesis driven). Case-specific monitoring may be considered necessary for GDMMs according to obligations under international treaties and/or national law and its logical basis in risk assessment as described therein. However, the need for general surveillance should be examined carefully; although it is a stated requirement in some countries, it may not be considered scientifically, technically, or financially justifiable beyond those jurisdictions. The expectation for general surveillance appears to be derived from a broad interpretation of the precautionary principle, which requires proof that an activity will not be harmful.^191^ Examples for pesticides and GMOs in the European Union (Supplemental File 1) suggest that general surveillance imposes considerable administrative burden but has provided little new information useful for decision-making. Moreover, a report sponsored by the EFSA concluded that standard methods of general surveillance will not be useful for PMEM of gene drive-modified insects.^74^

Although risk assessment conclusions based on credible pathways to harm provide primary input to monitoring plans, given the limited prior experience with gene drive technologies, relying solely on case-specific monitoring may be considered insufficient in some contexts. We, therefore, propose an approach that recognizes three possible monitoring categories from which ecological indicators may be selected:

• case-specific monitoring that targets pathways identified as having a plausible likelihood of being adversely impacted by the release of GDMMs, particularly those with residual uncertainty after risk assessment or where risk management/mitigation methods require additional validation;
• ecologically relevant monitoring for effects on biodiversity that focuses on priority indicator species as identified using a ranking approach; and
• culturally relevant monitoring that addresses species that may not fall within the first two categories but are of particular interest to communities where the GDMMs will be released.

Depending on the context, indicators could be selected from one or more of these categories. Such a “Goldilocks” approach (not too little and not too much) to monitoring would allow for identification of a manageable set of indicators that are broadly reflective of environmental and societal concerns, potentially offering more extensive coverage than case-specific monitoring alone but with choices being more rigorous and transparent than general surveillance. This science-based approach responds to the EFSA recommendation for design of alternatives to general surveillance for monitoring the unanticipated effects of gene drive-modified insects.^74^

### Adaptive monitoring allows for maximum responsiveness to ecosystem effects.

An adaptive approach to monitoring (Figures 1 and 3B) is recommended to facilitate timely response to evidence. Consideration of GDMM characteristics suggests that monitoring intensity should be greatest for 1) early-phase testing when there are no prior data on ecosystem interactions and 2) the period when genetic and entomological efficacy initially peaks after release and any resultant ecosystem interactions are expected to be most pronounced. Spatial scope and frequency of monitoring can be adjusted as more data are collected. Monitoring intensity also will be dictated by features of the selected indicator NTOs, including generation time, population size variability, and dispersal characteristics.

Precedents from classical biological control and SIT programs indicate that monitoring should not a priori be expected to continue perpetually, even when long-term and large-scale reduction in pest abundance or even eradication of the target pest species is the goal. Decisions about ending environmental monitoring can be based on evidence indicating a minimal risk of further ecological consequences, such as consistent absence of observable adverse effects on indicator NTOs and/or stable or declining gene drive population dynamics resulting from ecological or evolutionary processes. Conversely, the adaptive approach would favor continued monitoring if evidence suggests that risk is ongoing or expanded monitoring if an undesirable trend is suspected. Thus, a benefit of using an adaptive approach is the opportunity to base decisions about stopping, continuing, or expanding monitoring on data collected under local conditions.

## CONCLUSIONS: BEST PRACTICES FOR ENVIRONMENTAL MONITORING OF GDMMS ARE PROPOSED

With first field releases estimated to be only a few years away, developers and decision-makers will soon be required to develop plans for postrelease monitoring of GDMMs. At present, much more thought has gone into planning efficacy monitoring than safety monitoring. The recommendations for environmental safety monitoring presented here and summarized in Table 5 complement and extend prior guidance for testing and implementation of GDMMs,^9^^,^^26^^,^^28^ building on experience with other regulated products having relevant characteristics. Although it will be necessary for individual environmental monitoring plans to be based on characteristics of the particular GDMMs that will be released and the receiving environment, these recommendations advance a coherent framework to support preparations and provide assurance that safety monitoring for GDMMs can be advanced in a systematic and scientifically justifiable manner that addresses both environmental and societal interests.

**Table 5:** Summary of best-practice recommendations for environmental safety monitoring of gene drive-modified mosquitoes

Best-Practice Recommendations for Environmental Safety Monitoring
**Identify and engage stakeholders early** to support coordinated monitoring, including regulatory agencies, biosafety committees, health authorities, national vector control, and biodiversity programs
**Ensure transparency and inclusivity**, involving local communities and multidisciplinary experts in the development and refinement of monitoring plans and in reporting of results to ensure relevance and foster trust
**Adopt a risk-based approach** that determines on a case-by-case basis what constitutes unacceptable harm relative to environmental protection goals, significance of effects, and causal linkage to GDMM release
**Select indicators strategically**, considering case-specific (risk-based), ecologically relevant (ecosystem), and culturally relevant (community-valued) options that address diverse concerns and using a transparent ranking mechanism for prioritization
**Design proportionate monitoring plans** that balance risk management goals with available resources and timelines relevant to the selected indicators, avoiding unnecessary logistical and administrative burden from surveillance methods with limited decision-making value
**Clarify roles and responsibilities** of technology developers, permit holders, and regulators in the monitoring process
**Initiate early baseline ecological studies** leveraging existing vector or biodiversity monitoring platforms to assess interactions with the target vector species and understand intrinsic variability in indicator NTOs
**Integrate efficacy and ecological monitoring** for economies of scale, such as using the bycatch from entomological sampling to track changes in NTOs and high-throughput molecular methods for NTO identification
**Implement adaptive monitoring** using precedents from related technologies and predictive models to inform initial expectations for scale and duration and adjusting plans based on accumulated experience and observed effects

GDMM = gene drive-modified mosquito; NTO = nontarget organism. Key aspects of each recommendation are designated in bold.

## Supplemental Materials

10.4269/ajtmh.26-0049 Supplemental Materials
