# Supplementary material for: Considerations for Postrelease Environmental Safety Monitoring of Gene Drive-Modified Mosquitoes as a Tool for Malaria Control in Sub-Saharan Africa
Source: Am J Trop Med Hyg. 2026 Aug 4;115(4):660–82. doi: 10.4269/ajtmh.26-0049 (PMC13444447; doi:10.4269/ajtmh.26-0049)
Supplement: Supplementary file 1 [file tpmd260049.SD1.pdf]

Supplementary File 1. Review of environmental monitoring practices for relevant biological, chemical, and genetic technologies

While not exhaustive, this section provides an illustrative review of environmental monitoring requirements for other pest and vector control modalities with characteristics relevant to GDMMs.

Classical biological control

Classical biological control involves the importation and release of non-native natural enemies to manage pest organisms, most often invasive insects and weeds, that have appeared in new regions outside their native range.[1] Classical biological control has been practiced for over 100 years in many countries around the world. Because classical biological control programs involve the indefinite spread and persistence of a novel, self-propagating organism in the environment, concerns have been raised about the possibility of irreversible unintended ecological impacts.[2,3] While originally a trial-and-error practice with little attention to the potential for unintended consequences, biological control has undergone transformation over time to a carefully regulated pest management tactic informed by ecological principles.[1] Increased regulations and stricter standards still put most of the emphasis on risk, but there is now an effort towards a more balanced perspective that takes potential benefits (monetary, ecological, socioeconomic) into account.[4,5] Evaluation of risks remains largely conducted at the pre-release stage, collecting data to inform the risk assessment.[6] Post-release monitoring is recommended (although not required or enforced) in most jurisdictions, but there are no standardized requirements among countries as to the length of the monitoring period. International standards have been promulgated for the import and release of exotic biological control agents.[7] These call for importers to make information relating to the safety and environmental impact of biological control agents publicly available but there is no stated monitoring requirement. Biological control agents, including

invertebrates, that are able to cross national boundaries can become established in countries not participating in the original release, and a harmonized approach to regulation have been developed and partially adopted in both North American and European regions.[8,9] In North America, these standards state that a post-release monitoring plan should be part of the application for first release (RSPM 7 and 12), while in Europe post-release monitoring is encouraged (PM6/2 (4)). Neither region stipulates any detailed monitoring requirements.

Where described, case specific monitoring recommendations focus on following the establishment and spread of the biological control agent, its effect on the target species, and any direct effects on NTOs.[10,11,12] The potential for negative indirect ('knock-on') ecological effects is now routinely considered in pre-release risk assessments but is extremely challenging to monitor in practice, and post-release monitoring has documented indirect ecological effects only in a very limited number of cases.[5,12,13]

Examples of ongoing monitoring of populations of NTOs over space and time after classical biological control releases are very rare, and most post-release monitoring studies involve retrospective observations of NTO status performed years after release of the biological control agent.[14,15,16] Monitoring data has occasionally been collected within the same year(s) as releases when it was required in the release permit.[17] Notably, the most useful insights about biological control impacts on NTOs have been gained from examination of specific ecological hypotheses by voluntary research studies working on limited spatial and temporal scales, outside the context of formal post-release monitoring requested by regulators.[13]

Chemical pesticides

Despite the yearly application of billions of kilograms of pesticides, insect pests destroy approximately 14% of all potential food production,[18] with global losses substantially greater among certain crops.[19] Many of these chemical insecticides demonstrate broad spectrum toxicity, including not only for multiple insect species but also for humans and animals.[20] In the USA, approximately 50% of pesticide applications are via aircraft, resulting in widespread dissemination and higher NTO exposure.[21] Regulatory decisions about pesticide registration generally are based on risk assessments, using data provided by pre-release toxicity testing.[22, 23] Although not required, it has been deemed desirable to examine, at some time after introduction, whether the environmental effects of the specific pesticide have been adequately predicted by risk assessment.[24,25] For example, in some jurisdictions there is monitoring of pesticide residue levels in water, on food, or in other parts of the environment.[26-29] In the EU, examination of environmental contamination with three previously approved neonicotinoids was judged to pose a risk to bees, which resulted in a ban of these pesticides for outdoor uses in the European Union.[30] Monitoring of effects on NTOs is also suggested but not required.[24,25] A rare example of general surveillance is the program undertaken by the French Ministry of Agriculture to assess the unintended effects of agricultural practices, including pesticide use, on biodiversity.[31] Between 2013 and 2016 (4 years), over 12,000 biodiversity surveys were conducted across metropolitan France and Corsica, including multiple taxa of NTOs (14 groups of beetles, 31 species of birds, 151 species of flora, and 8 groups of earthworms). For most taxa, four years of large-scale surveys were insufficient to detect significant changes in long-term trends in species abundance or community composition. However, a recent meta-analysis of some 1700 experimental studies confirmed widespread negative effects of pesticides on terrestrial and aquatic NTOs.[32]

In addition to agricultural uses, chemical pesticides are important public health tools. Control of the mosquito vectors of malaria currently depends overwhelmingly on use of insecticides. While insecticide-based mosquito control is responsible for saving millions of lives,[33] it is accompanied by ongoing problems with insecticide resistance as well as increasing evidence of environmental and health impacts.[34] DDT, an organochlorine pesticide with high persistence and bioaccumulation potential,[35] is still used to control malaria in some African countries despite proven harm for apex predators.[36] Although pyrethroid pesticides used for malaria control are far more toxic to insects than other groups of organisms,[37-39] adverse effects on non-target vertebrates such as lizards,[40] fish, and other non-target aquatic organisms,[41,42] as well as humans[43] have been documented.

A review of the African pesticide registration landscape conducted in 2019 revealed no uniform dossier requirements for chemical vector control products;[44] work is underway, however, to pilot the WHO Collaborative Registration procedure to facilitate national decisions for marketing authorizations.[45,46] The WHO has a prequalification process for public health pesticides that takes hazard exposure as well as acute toxicity to animals into account.[47] The two most frequently used control methods for *Anopheles* vectors of malaria, long-lasting insecticide treated nets and indoor residual spraying, are intended to limit NTO exposure to insecticides. Risk considerations for these methods has focused on safety to human health, with little attention to ecosystem effects.[48,49] However, recent studies suggest they can have toxic effects on other domiciliary pests and may stimulate insecticide resistance in these species.[50] While an International Code of Conduct states that there should be provision for a mechanism for reporting longer term adverse effects on health and the environment caused by pesticides as well as for collecting and analyzing such data,[25] this is not included in the WHO prequalification process and is left to national governments to execute.

Other vector control tools

Sterile Insect Technique involves ongoing inundative releases of living chemical or radiation-sterilized male insects, which mate unproductively with local females of the same species, thereby reducing numbers of the target pest insect species over time. Among 36 projects testing SIT alone or in combination with *Wolbachia*-mediated sterility (i.e., Incompatible Insect Technique), to suppress *Aedes* mosquitoes,[51] none were required by regulators to conduct environmental monitoring because their action via mating is understood to confer species specificity (J. Bouyer, personal observation). In an SIT program for tsetse eradication in Senegal, however, a monitoring plan was provided and approved by the program's management at the request of the Ministry of the Environment. Annual assessments of indicator species have thus far been conducted over thirteen years in five sampling locations, providing an example of long-term data collection.[52] Boosted SIT is a newer technology in which sterile males are treated with a biocide before release[53] and transfer the biocide to females during mating or to larval habitats, thus extending efficacy. Small-scale boosted SIT field trials have been conducted against *Aedes albopictus* in Spain and *Aedes aegypti* in Reunion island.[54] In Reunion, the regional health agency requested monitoring of the impact of the technology against NTOs. Monitoring in treated and control sites of the abundance and emergence rate of chironomids (midges) sharing the same habitats as the target mosquitoes as well as of the health of beehives[55] showed no significant effect. For future scaled-up field trials, the regional Direction of the Environment has requested that, in addition to these two NTO groups, other species sharing the larval habitats of *Aedes* mosquitoes be monitored. In parallel to standard monthly taxonomical monitoring of NTOs in 45 sentinel larval habitats for 2 years, biodiversity of bacteria and arthropods will be monitored using eDNA and metagenomic techniques.

Limited environmental monitoring has been conducted for other mosquito control tools. A Kenyan study[56] of mosquito larval habitats for the impact of long-lasting microbial larvicides monitored the

larval abundance of 11 taxa of NTOs in 3 highland villages for 5 months. A study of toxic sugar bait stations containing a pesticide and dye marker, conducted in houses in 5 villages in Mali, involved 3 months of daily monitoring to compare effects on adult NTOs that either had or had not fed at the stations, as detected by presence of marker dye.[57] In both studies, duration of monitoring correlated with the anticipated duration of the intervention strategy.

The bacterial endosymbiont *Wolbachia* has been applied for control of mosquito vectors in two ways. A population suppression approach relies on inundative release of *Wolbachia*-infected male *Aedes* mosquitoes to mate unproductively with local females, with a result similar to SIT (the Incompatible Insect approach). This method has been approved for commercialization without requirement for post-marketing environmental monitoring.[58,59] A population modification approach, which blocks replication of arboviruses within the mosquito, is based on release of both male and female *Aedes aegypti* mosquitoes infected with *Wolbachia*. With this method, infected females preferentially pass *Wolbachia* to their progeny thereby increasing its frequency in the local *Aedes* population, constituting a natural (non-GMO) drive. In this case, *Wolbachia* has been documented to remain stable at high frequency in *Aedes aegypti* populations for at least 7 years.[60] Risk assessments have led several countries to conclude that these *Wolbachia*-infected mosquitoes pose a negligible risk of causing more harm than their uninfected counterparts over a 30 year period.[61-63]

Other GMOs

The first large-scale commercialization of GM plants was reported in the USA in 1996.[64] Since then, several other GM agricultural products have been commercialized (soybean, maize, cotton and canola), constituting 47% of global planting in 2020.[64] A 2001 European Commission resolution made post release monitoring of GMOs mandatory.[65] Resulting guidance for post-marketing environmental

monitoring (PMEM) of GM plants by the European Food Safety Authority (EFSA)[66] addresses both case-specific and general monitoring as well as spatial and temporal scale, recommending monitoring at sites of greatest exposure over a time period sufficient to detect potential delayed adverse effects. A few countries have since adopted similar guidelines for post-release monitoring but with limited reporting on their experiences (e.g., Brazil[67] and Kenya[68]).

A widely cited example is the cultivation of GM maize producing an insecticidal protein originating from the bacterium *Bacillus thuringiensis* (MON 810), which was authorized under Directive 90/220/EEC in the European Union by the Commission Decision 98/294/EC.1.[69] PMEM of GM maize has largely focused on maintenance of efficacy, i.e. detection of resistance to the insecticidal protein in targeted pest species. Since 2003, the permit holder has followed a harmonized insect resistance management plan,[70,71] which focuses on a high-dose/refuge strategy.[72] Periodic monitoring is typically conducted to evaluate the adequacy and effectiveness of the strategy. This approach relies on case specific monitoring for signs of tolerance to the introduced insecticidal *Bt* protein in field populations of the target pest using laboratory bioassays, as well as farmers' reports of product-related issues such as loss of efficacy. General surveillance activities, intended to detect unanticipated adverse effects, include surveys based on questionnaires from farmers and systematic literature searches to find relevant scientific publications.[73,74] Farmers are required to indicate changes to several environmental parameters (negative, positive, or unchanged) in comparison to a conventional crop. However, it is unclear how changes, if detected, would be causally attributed to the GM crop and considered for case-specific specific monitoring. The permit holder reports the results of monitoring for resistance evolution and refuge compliance, and general surveillance, to the European Commission and the EU Member States. EFSA evaluation of MON810 annual reports from 2009-2022 concluded continuing sufficiency of existing risk assessment and risk management recommendations based on the lack of new hazards or

new scientific uncertainties.[74] In a related study, statistical analysis of pooled farmer questionnaires from 2006-2015 also failed to reveal any associated unexpected adverse effects.[75]

A survey of regulatory requirements in 10 African countries with the most developed biosafety laws all mention post-marketing monitoring as a requirement but provide few specifics on how, when, by whom, and for how long.[76] Requirements vary greatly depending on country. Kenya, for example, requires both case-specific and general monitoring of GM crops via a PMEM plan developed by the applicant and approved by the competent authority.[68] The Kenyan National Biosafety Authority requires annual reporting for 10 years with a further 10-year requirement upon permit renewal. Monitoring sites should be representative of different ecosystems exposed to the GMO over time with comparison pre- and post-GMO exposure. Some countries require strategic environmental assessment and/or environmental and social impact assessments for new development projects to capture their potential socioeconomic effects and this may be applicable to GDMMs.[77] These may require a monitoring system to ensure that management practices for mitigating any negative impacts are followed.[78]

Guidance for GM animals, including insects, has likewise been published by EFSA, which recommends case-specific PMEM when there are important information or knowledge gaps, insufficient risk mitigation/management, or significant levels of risk identified in the environmental risk assessment.[66] This guidance considers the need for general surveillance to detect unanticipated adverse effects, and suggests this might be accomplished through literature reviews, questionnaires to breeders/producers, or existing monitoring networks such as veterinary inspection services. It does not discuss the extent to which this might be applicable to insects and merely states that it is the task of the applicant to identify appropriate tools for general surveillance. A GM variation on SIT, developed for suppression of *Aedes aegypti* vectors of arboviral diseases, relies on a "Tet-Off" system, a conditional lethality system in which the modification is silenced in the presence of tetracycline.[79] In the prototype strain (OX513A) the

modification prevents development of viable male and female progeny upon mating with local wild type females in the absence of tetracycline.[80] Like classical SIT, the population suppression stops once releases end. Initial post-release monitoring mandated by the Brazilian competent authority (CTNBio) for OX513A mosquitoes required stability assessment of the GM construct using its encoded marker gene every month for the first year and on an annual basis thereafter, examination of tetracyline use around wastewater plants and via analysis of literature and research reports, and measurement of *Aedes albopictus* numbers in selected areas to address the potential for niche replacement and competitive release of a related vector species.[81] There were no other ongoing PMEM requirements for the product. This technology has now transitioned to a subsequent strain (OX5034), in which only development of female progeny is inhibited. In this strain, the male-selecting trait is reported to persist in the population at progressively diminishing levels for approximately 10 generations.[82] This is the only registered GM mosquito product currently in use, although a similar construct in *An. stephensi* is being trialed in Djibouti,[82] where the Ministry of Housing, Urban Planning, Environment and Regional Development is the Competent National Authority for regulatory approval.[84]

A further report examining the adequacy and sufficiency of existing EFSA guidelines for PMEM of GM insects containing engineered gene drives recognizes that general surveillance is not specifically targeted at particular indicators relevant to either assumptions in the environmental risk assessment or to some specific harm to the environment, and therefore is not useful in the PMEM of gene drive-modified insects. This report concludes with a recommendation that further consideration is needed for the design and implementation of methods to identify potential unanticipated adverse effects in a proportionate manner.[85]

References, Supplementary File 1

1. Heimpel GE, Mills NJ. *Biological Control: Ecology and Applications*. Cambridge University Press; 2017.
2. Howarth FG. Classical Biocontrol: Panacea or Pandora's Box. *Proc Hawaii Entomol Soc*. 1983;24(2 & 3):239-244.
3. Simberloff D, Stiling P. How Risky is Biological Control? *Ecology*. 1996;77(7):1965-1974. https://doi.org/10.2307/2265693 Accessed January 27, 2026
4. Abram PK, Franklin MT, Brodeur J, Cory JS, McConkey A, Wyckhuys KAG, Heimpel GE. Weighing consequences of action and inaction in invasive insect management. *One Earth*. 2024;7(5):782-793. https://doi.org/10.1016/j.oneear.2024.04.013 Accessed January 27, 2026
5. Heimpel GE, Abram PK, Causton CE, Cells SL, Coll M, Hardy ICW, Mangel M, Mills NJ, Segoli M. A benefit–risk analysis for biological control introductions based on the protection of native biodiversity. *Ecol Appl*. 2024;34(6):e3012. https://doi.org/10.1002/eap.3012 Accessed January 27, 2026
6. Bigler F, Babendreier D, Kuhlmann U. Environmental Impact of Invertebrates for Biological Control of Arthropods: Methods and Risk Assessment. Vol 10. CABI; 2006. https://www.cabidigitallibrary.org/doi/book/10.1079/9780851990583.0000 Accessed July 13, 2026
7. FAO. International Standards for Phytosanitary Measures: Code of Conduct for the Import and Release of Exotic Biological Control Agents. Food and Agriculture Organization; 1996;23. https://www.ippc.int/largefiles/adopted_ISPMs_previousversions/en/ISPM_03_1995_En_1998-07-13.pdf Accessed July 13, 2026
8. EPPO. PM6 Standards on safe use of biological control. 2025. EPPO Standards on safe use of biological control Accessed July 13, 2026

9. NAPPO. Regional Standards for Phytosanitary Measures (RSPM) :: nappo.org. 2025. https://www.nappo.org/english/products/regional-standards-phytosanitary-measures-rspm Accessed July 13, 2026
10. NAPPO. NAPPO Regional Standards for Phytosanitary Measures (RSPM), RSPM 12. North American Plant Protection Organization 2015;14. https://www.nappo.org/application/files/4015/8352/2959/RSPM_12-30-07-15-e.pdf Accessed July 13, 2026
11. NAPPO. NAPPO Regional Standards for Phytosanitary Measures (RSPM), RSPM 7. 2015;13. https://www.nappo.org/application/files/5315/8352/2957/RSPM_07-30-07-15-e.pdf Accessed July 13, 2026
12. Henneman ML, Memmott J. Infiltration of a Hawaiian Community by Introduced Biological Control Agents. *Science*. 2001;293(5533):1314-1316. Infiltration of a Hawaiian Community by Introduced Biological Control Agents | Science Accessed January 27, 2026
13. Heimpel GE, Cock MJW. Shifting paradigms in the history of classical biological control. *BioControl*. 2017;63(1):27-37. https://doi.org/10.1007/s10526-017-9841-9 Accessed January 27, 2026
14. Barratt BIP, Todd JH, Malone LA. Selecting non-target species for arthropod biological control agent host range testing: Evaluation of a novel method. *Biol Control*. 2016;93:84-92. https://doi.org/10.1016/j.biocontrol.2015.11.012. Accessed January 27, 2026
15. Lynch LD, et al. Insect Biological Control and Non-target Effects : a European Perspective. In: Evaluating Indirect Ecological Effects of Biological Control. CABI; 2001;99-125. https://research.wur.nl/en/publications/insect-biological-control-and-non-target-effects-a-european-persp Accessed January 27, 2026

16. Barratt BIP, Blossey B, Hokkanen HMT. Post-release evaluation of non-target effects of biological control agents. In: Environmental Impact of Invertebrates for Biological Control of Arthropods: Methods and Risk Assessment. CABI Books. 2006;166-186. https://doi.org/10.1079/9780851990583.0166 Accessed January 27, 2026
17. Fellin L, Grassi A, Puppato S, Saddi A, Anfora G, Ioriatti C, Rossi-Stacconi MV. First report on classical biological control releases of the larval parasitoid *Ganaspis brasiliensis* against *Drosophila suzukii* in northern Italy. *BioControl*. 2023;68(1):1-12. https://doi.org/10.1007/s10526-022-10174-2 Accessed January 27, 2026
18. Pimentel D. Area-Wide Pest Management: Environmental, Economic and Food Issues. In: Springer, Dordrecht. 2007;35-47. https://doi.org/10.1007/978-1-4020-6059-5_2 Accessed January 27, 2026
19. Oerke EC. Crop losses to pests | The Journal of Agricultural Science. *J Agric Sci*. Published online 2005. https://doi.org/10.1017/S0021859605005708 Accessed July 13, 2026
20. Roberts JR, Reigart JR. Recognition and Management of Pesticide Poisonings: Sixth Edition: 2013. Environmental Protection Agency. 2013;277. https://www.epa.gov/sites/default/files/2015-01/documents/rmpp_6thed_final_lowresopt.pdf Accessed July 13, 2026
21. Pimentel D, Burgess M. Small amounts of pesticides reaching target insects. *Environ Dev Sustain*. 2011;14(1):1-2. https://doi.org/10.1007/s10668-011-9325-5 Accessed January 27, 2026
22. EPA. Data Requirements for Pesticide Registration. United States Environmental Protection Agency. April 13, 2026. https://www.epa.gov/pesticide-registration/data-requirements-pesticide-registration#ef Accessed January 27, 2026

23. WHO. The WHO Recommended Classification of Pesticides by Hazard and guidelines to classification, 2019 edition. https://www.who.int/publications/i/item/9789240005662 Accessed July 13, 2026
24. FAO. Guidelines on Post-Registration Surveillance and Other Activities in the Field of Pesticides. Food and Agriculture Organization. 1988;14. https://openknowledge.fao.org/server/api/core/bitstreams/19de82ba-21b8-4a3c-9ac6-2a66c35870f0/content Accessed July 13, 2026
25. WHO. Guidelines for the Registration of Pesticides. International Code of Conduct on the Distribution and Use of Pesticides. World Health Organization. 2010;42. https://www.who.int/publications/i/item/who-htm-ntd-whopes-2010.7 Accessed July 13, 2026
26. Baker NT, Wilson JT, Moran MJ. Methods and Sources of Data Used to Develop Selected Water–Quality Indicators for Streams and Ground Water for EPA's 2007 Report on the Environment: Science Report. 2008;23. https://pubs.usgs.gov/of/2008/1096/ Accessed January 27, 2026
27. CFIA. National Chemical Residue Monitoring Program (NCRMP) Report from 2021 to 2022. Canadian Food Inspection Agency. 2024. https://inspection.canada.ca/en/food-safety-industry/food-chemistry-and-microbiology/testing-reports-and-journal-articles/2021-2022-ncrmp-report Accessed July 13, 2026
28. Fourcade M, Gervais V, Simon-Delavelle F, Lavarde P, Veerabadren A, Joly A. *Prévenir et maîtriser les risques liés à la présence de pesticides et de leurs métabolites dans l'eau destinée à la consommation humaine*. Republique Francaise. 2024;586. https://www.vie-publique.fr/files/rapport/pdf/296319.pdf Accessed January 27, 2026
29. Gomez Cortes L, Porcel Rodriguez E, Marinov D, Sanseverino I, Lettieri T. Selection of Substances for the 5th Watch List under the Water Framework Directive. Publications Office of the European Union. 2025. https://data.europa.eu/doi/10.2760/956398 Accessed July 13, 2026

30. EU. Neonicotinoids - European Commission. European Commission. Published online 2025. https://food.ec.europa.eu/plants/pesticides/approval-active-substances-safeners-and-synergists/renewal-approval/neonicotinoids_en Accessed July 13, 2026
31. Andrade C, et al. A real-world implementation of a nationwide, long-term monitoring program to assess the impact of agrochemicals and agricultural practices on biodiversity. *Ecol Evol*. 2021;11(9):3771-3793. https://doi.org/10.1002/ece3.6459 Accessed January 27, 2026
32. Wan NF, et al. Pesticides have negative effects on non-target organisms. *Nat Commun*. 2025;16(1):1360. https://doi.org/10.1038/s41467-025-56732-x Accessed January 27, 2026
33. Kleinschmidt I, et al. Implications of insecticide resistance for malaria vector control with long-lasting insecticidal nets: a WHO-coordinated, prospective, international, observational cohort study. *Lancet Infect Dis*. 2018;18(6):640-649. https://doi.org/10.1016/S1473-3099(18)30172-5 Accessed January 27, 2026
34. Knols BG. A shot in the foot: Could chemical control of malaria vectors threaten food security? *MalariaWorld J*. 2024;15(13):1-7. https://doi.org/10.5281/zenodo.13969756 Accessed January 27, 2026
35. Jayaraj R, Megha P, Sreedev P. Review Article. Organochlorine pesticides, their toxic effects on living organisms and their fate in the environment. *Sciendo*. Published online 2017. https://doi.org/10.1515/intox-2016-0012 Accessed January 28, 2026
36. Makgoba L, Abrams A, Röösli M, Cissé G, Dalvie MA. DDT contamination in water resources of some African countries and its impact on water quality and human health. Published online April 15, 2024. https://www.cell.com/heliyon/abstract/S2405-8440(24)04085-4 Accessed January 28, 2026

37. Ahamad A, Kumar J. Pyrethroid pesticides: An overview on classification, toxicological assessment and monitoring. *J Hazard Mater Adv*. 2023;10:100284. https://doi.org/10.1016/j.hazadv.2023.100284 Accessed January 28, 2026
38. Krueger AJ, Hanford K, Weissling TJ, Vélez AM, Anderson TD. Pyrethroid Exposure Reduces Growth and Development of Monarch Butterfly (Lepidoptera: Nymphalidae) Caterpillars. *J Insect Sci*. 2021;21(2):2. https://doi.org/10.1093/jisesa/ieaa149 Accessed January 28, 2026
39. Liu Q, He Q, Zhang S, Chai Y, Gao Q, Xiao J, Fang Q, Yu L, Cao H. Toxic effects of detected pyrethroid pesticides on honeybee (*Apis mellifera ligustica* Spin and *Apis cerana cerana* Fabricius). *Sci Rep*. 2022;12:16695. https://doi.org/10.1038/s41598-022-20925-x Accessed July 13, 2026
40. Yermolenko SV, Gasso VY, Spirina VA, Huslystyi AO. Influence of pyrethroid and neonicotinoid insecticides on lizards (Reptilia, Squamata) (a review). *Oles Honchar Dnipropetrovsk Natl Univ*. Published online 2021. https://doi.org/10.15421/442109 Accessed January 28, 2026
41. Li L, et al. The toxicokinetics and risk assessment of pyrethroids pesticide in tilapia (*Oreochromis mossambicus*) upon short-term water exposure. *Ecotoxicol Environ Saf*. 2022;241:113751. https://doi.org/10.1016/j.ecoenv.2022.113751 Accessed January 28, 2026
42. Ranatunga M, Kellar C, Pettigrove V. Toxicological impacts of synthetic pyrethroids on non-target aquatic organisms: A review. *Environ Adv*. 2023;12:100388. https://doi.org/10.1016/j.envadv.2023.100388 Accessed January 28, 2026
43. Ramchandra AM, Chacko B, Victor PJ. Pyrethroid Poisoning. *Indian J Crit Care Med*. 2019;23(Suppl 4):S267-S271. https://doi.org/10.5005/jp-journals-10071-23304 Accessed January 28, 2026
44. Innovation to Impact. Country Registration. 2025. Accessed November 14, 2025. https://innovationtoimpact.org/workstreams/country-registration/ Accessed July 13, 2026

45. Innovation to Impact. Launch of the WHO Collaborative Registration Procedure for Vector Control Products pilot scheme. 2025. https://innovationtoimpact.org/launch-of-who-collaborative-registration-procedure-for-vector-control-products-workshop-2024/ Accessed July 13, 2026

46. WHO. Collaborative Registration Procedure for prequalified Vector Control Products. Regulation and Prequalification. 2025. Accessed November 14, 2025. https://www.who.int/teams/regulation-prequalification/regulation-and-safety/facilitated-product-introduction/collaborative-registration-procedure/crp-for-prequalified-vector-control-products Accessed July 13, 2026

47. WHO. Overview of the WHO Prequalification Assessment of Vector Control Products. World Health Organization. 2021;31. https://extranet.who.int/prequal/sites/default/files/document_files/WHO_PQT_VectorControlProducts_June2021.pdf Accessed July 13, 2026

48. WHO. *Generic Risk Assessment Model for Insecticide-Treated Nets*. World Health Organization. 2018;54. https://www.who.int/publications/i/item/9789241513753 Accessed January 28, 2026

49. WHO. Generic Risk Assessment Model for Indoor Residual Spraying of Insecticides. 2nd ed. World Health Organization. 2018. https://iris.who.int/handle/10665/260549 Accessed July 13, 2026

50. Hayes CC, Schal C. Review on the impacts of indoor vector control on domiciliary pests: good intentions challenged by harsh realities. *Proc R Soc B Biol Sci*. 2024;291(2027):20240609. https://doi.org/10.1098/rspb.2024.0609 Accessed January 28, 2026

51. Bouyer J. Current status of the sterile insect technique for the suppression of mosquito populations on a global scale. *Infect Dis Poverty*. 2024;13(1):68. https://doi.org/10.1186/s40249-024-01242-z Accessed January 28, 2026

52. Ciss M, Bassène MD, Seck MT, Mbaye AG, Sall B, Fall AG, Vreysen MJB, Bouyer J. Environmental impact of tsetse eradication in Senegal. *Sci Rep*. 2019;9(1):20313. https://doi.org/10.1038/s41598-019-56919-5 Accessed January 28, 2026
53. Bouyer J, Lefrançois T. Boosting the sterile insect technique to control mosquitoes. *Trends Parasitol*. 2014;30(6):271-273. https://doi.org/10.1016/j.pt.2014.04.002 Accessed January 28, 2026
54. Bouyer J, et al. Suppression of *Aedes* mosquito populations with the boosted sterile insect technique in tropical and Mediterranean urban areas. *Sci Rep*. 2025;15(1):17648. https://doi.org/10.1038/s41598-025-02795-1 Accessed January 28, 2026
55. Rao Z, Cao L, Wu H, Qiu X, Liu G, Han R. Comparative Transcriptome Analysis of Thitarodes Armoricanus in Response to the Entomopathogenic Fungi Paecilomyces Hepiali and Ophiocordyceps Sinensis. *Insects*. 2019;11(1):4. https://doi.org/10.3390/insects11010004 Accessed January 28, 2026
56. Derua YA, hindi SC, Mosha FW, Kweka EJ, Atieli HE, Wang X, Zhou G, Lee M-C, Githeko A, Yan G. Microbial larvicides for mosquito control: Impact of long lasting formulations of Bacillus thuringiensis var. israelensis and Bacillus sphaericus on non-target organisms in western Kenya highlands. *Ecol Evol*. 2018;8(15):7563-7573. https://doi.org/10.1002/ece3.4250 Accessed January 28, 2026
57. Diarra RA, et al. Testing configurations of attractive toxic sugar bait (ATSB) stations in Mali, West Africa, for improving the control of malaria parasite transmission by vector mosquitoes and minimizing their effect on non-target insects. *Malar J*. 2021;20(1):184. https://doi.org/10.1186/s12936-021-03704-3 Accessed January 28, 2026
58. EPA. The *Wolbachia* bacterium prevents the development of Yellow Fever (*Aedes aegypti*) mosquito eggs. Published online 2024.

https://www3.epa.gov/pesticides/chem_search/ppls/089668-00008-20240416.pdf Accessed July 13, 2026

59. Mains JW, Kelly PH, Dobson KL, Petrie WD, Dobson SL. Localized control of *Aedes aegypti* (Diptera: Culicidae) in Miami, FL, via inundative releases of *Wolbachia*-infected male mosquitoes. *J Med Entomol*. 2019;56(5):1296-1303. https://doi.org/10.1093/jme/tjz051 Accessed January 28, 2026
60. Ross PA, et al. A decade of stability for wMel *Wolbachia* in natural Aedes aegypti populations. *PLoS Pathog*. 2022;18(2):e1010256. https://doi.org/10.1371/journal.ppat.1010256 Accessed January 28, 2026
61. Buchori D, Aryati D, Hadi UK, Joseph HK. Risk Assessment on the Release of *Wolbachia*-Infected *Aedes aegypti*. World Mosquito Program. 2017;79. https://www.worldmosquitoprogram.org/sites/default/files/2019-12/risk_assessment_on_the_release_of_wolbachia-infected_aedes_aegypti.pdf Accessed July 13, 2026
62. Hoc TQ, Uyen Ninh T, Tuat NV, Hung NV, Cuong ND. *Risk Assessment of the Pilot Release of Aedes Aegypti Mosquitoes Containing Wolbachia*. World Mosquito Program. 2011;69. https://www.worldmosquitoprogram.org/sites/default/files/july_2011_ra_report_eng.pdf Accessed July 13, 2026
63. Murphy B, Jansen C, Murray J, De Barro P. Risk Analysis on the Australian Release of *Aedes aegypti (L.) (Diptera: Culicidae)* Containing *Wolbachia.* CSIRO. 2010;109. https://www.worldmosquitoprogram.org/en/learn/scientific-publications/risk-analysis-australian-release-aedes-aegypti-l-diptera-culicidae Accessed July 13, 2026

64. Brookes G. Genetically Modified (GM) Crop Use 1996–2020: Environmental Impacts Associated with Pesticide Use Change. *GM Crops Food*. 2022;13(1):262-289. https://doi.org/10.1080/21645698.2022.2118497 Accessed January 28, 2026
65. EU Directive 2001/18/EC of the European Parliament and of the Council of 12 March 2001 on the deliberate release into the environment of genetically modified organisms and repealing Council Directive 90/220/EEC. Published online 2001. https://faolex.fao.org/docs/pdf/eur34933.pdf Accessed July 13, 2026
66. EFSA. Guidance on the Post-Market Environmental Monitoring (PMEM) of genetically modified plants. *EFSA J*. 2011;9(8):2316. https://doi.org/10.2903/j.efsa.2011.2316 Accessed January 28, 2026
67. Melo MA, Kido EA, Andrade PP. Post Market Monitoring: Legal Framework in Brazil and First Results(Contribution to the 4th International Workshop on PMEM of Genetically Modified Plants, Quedlinburg). *J Cultiv Plants*. Published online July 1, 2011:226-231 Pages. https://doi.org/10.5073/JFK.2011.07.04 Accessed July 13, 2026
68. NBA. Manual for post release monitoring of genetically modified crops in Kenya. Published online July 2021. https://www.biosafetykenya.go.ke/wp-content/uploads/2025/10/Approved-Manual-Post-Release-Monitoring.pdf Accessed July 13, 2026
69. EU Decision - 98/294 - EN - EUR-Lex. 98/294/EC: Commission Decision of 22 April 1998 concerning the placing on the market of genetically modified maize (Zea mays L. line MON 810), pursuant to Council Directive 90/220/EEC (Text with EEA relevance). 1998. https://eur-lex.europa.eu/eli/dec/1998/294/oj/eng Accessed January 28, 2026
70. Alacalde E, et al. Insect Resistance Monitoring for Bt Maize Cultivation in the EU: Proposal from the Industry IRM Working Group. *J Für Verbraucherschutz Leb*. 2008;2(1):47-49. https://doi.org/10.1007/s00003-007-0236-1 Accessed January 28, 2026

71. García M, García-Benítez C, Ortego F, Farinós GP. Monitoring Insect Resistance to Bt Maize in the European Union: Update, Challenges, and Future Prospects. *J Econ Entomol*. 2023;116(2):275-288. https://doi.org/10.1093/jee/toac154 Accessed January 28, 2026
72. Tabashnik BE, Brévault T, Carrière Y. Insect resistance to Bt crops: lessons from the first billion acres. *Nat Biotechnol*. 2013;31(6):510-521. https://doi.org/10.1038/nbt.2597 Accessed January 28, 2026
73. Schmidt K, Wilhelm R, Schmidtke J, Beisner L, Monkemeyer W, Bottinger P, Sweet J, Schiemann J. Farm questionnaires for monitoring genetically modified crops: a case study using GM maize. *Environ Biosafety Res*. 2008;7(3):163-179. https://doi.org/10.1051/ebr:2008015 Accessed January 28, 2026
74. EFSA, Messéan A, Álvarez F, Devos Y, Camargo AM. Assessment of the 2022 post-market environmental monitoring report on the cultivation of genetically modified maize MON 810 in the EU. *EFSA J*. 2024;22(8):e8986. https://doi.org/10.2903/j.efsa.2024.8986 Accessed January 28, 2026
75. Bertho L, Schmidt K, Schmidtke J, Brants I, Canton RF, Novillo C, Head G. Results from ten years of post-market environmental monitoring of genetically modified MON 810 maize in the European Union. Published online April 24, 2020. https://doi.org/10.1371/journal.pone.0217272 Accessed January 28, 2026
76. Njagi J, Muriuki J, Mbugua P, Dass B, James S and Ogoyi D. Regulatory provisions for post-release monitoring of genetically modified organisms in Africa. Front Bioeng Biotechnol. 2026; 14:1785298. https://doi.org/10.3389/fbioe.2026.1785298
77. Connolly JB, et al. Recommendations f/r environmental risk assessment of gene drive applications for malaria vector control. *Malar J*. 2022;21:152. https://doi.org/10.1186/s12936-022-04183-w Accessed January 28, 2026

78. Mozambique LNG. *ESHIA Executive Summary and Update*. Mozambique LNG. 2019;130. https://www.afdb.org/sites/default/files/documents/environmental-and-social-assessments/mozlng_eshia_executive_summary_and_update_compressed.pdf Accessed January 28, 2026

79. Phuc HK, et al. Late-acting dominant lethal genetic systems and mosquito control. *BMC Biol*. 2007;5(1):11. https://doi.org/10.1186/1741-7007-5-11 Accessed January 28, 2026

80. Oxitec. Oxitec: Technology. Oxitec. 2025. https://www.oxitec.com/en/our-technology#about-our-insects Accessed January 28, 2026

81. CTNBio. *Technical Opinion No.3964/2014*. Brazil National Biosafety Technical Commission; 2014:14. https://ctnbio.mcti.gov.br/api/media/file/thecnical_report_3964_2014_commercial_release_of_strain_ox513a_of_aedes_aegypti_process_01200.002919_2013_77.pdf Accessed July 13, 2026

82. Spinner SAM, et al. New self-sexing *Aedes aegypti* strain eliminates barriers to scalable an d sustainable vector control for governments and communities in dengue-prone environments. *Front Bioeng Biotechnol*. 2022;10-2022. https://doi.org/10.3389/fbioe.2022.975786 Accessed January 28, 2026

83. Oxitec. Oxitec: Djibouti Friendly™ Mosquito Program. Djibouti Mosquito Project. 2025. Accessed May 12, 2025. https://www.djiboutifriendlymosquito.com Accessed January 28, 2026

84. BCH. Djibouti | Country Profile | Biosafety Clearing-House. Biosafety Clearing-House. 2025. https://bch.cbd.int/en/countries/DJ/CNA Accessed January 28, 2026

85. EFSA, Naegeli H, Dalmay T, et al. Adequacy and sufficiency evaluation of existing EFSA guidelines for the molecular characterisation, environmental risk assessment and post-market environmental monitoring of genetically modified insects containing engineered gene drives.

EFSA J. 2020;18(11):e06297. https://doi.org/10.2903/j.efsa.2020.6297 Accessed January 28, 2026

Supplementary File 2. Information sources for hypothetical PRONTI scoring of biodiversity indicators

1. These references were used to inform identification of potential species interactions with *An. gambiae* in sub-Saharan Africa for generation of Figure 2A.

   - Collins, C.M., Bonds, J.A.S., Quinlan, M.M., & Mumford, J.D. (2019). Effects of the removal or reduction in density of the malaria mosquito, s.l., on interacting predators and competitors in local ecosystems. *Medical and Veterinary Entomology*, *33*(1), 1–15. https://doi.org/10.1111/mve.12327
   - Gimonneau, G., Bouyer, J., Morand, S., Besansky, N.J., Diabate, A., Simard, F.. (2010). A behavioral mechanism underlying ecological divergence in the malaria mosquito Anopheles gambiae. *Behavioral Ecology*, 21(5):1087-1092. https://doi.org/10.1093/beheco/arq114
   - Ouedraogo, J., Sawadogo, S.P., Niang, A.. Soulama, A., Yerbanga, S., Ouedraogo, T.F.X., Guissou, C., Dabire, R.K., Muller, R., Gnandine, O., & Diabate, A. (2025). Interaction of predatory macroinvertebrate communities with malaria vectors in aquatic habitats of three climatic zones in Burkina Faso. *Parasites & Vectors* **18**, 158 (2025). https://doi.org/10.1186/s13071-025-06794-z
   - Mahenge, H.H., Muyaga, L.L., Nikya, J.D., Kafwenji, A.D., Mwalugelo, Y.A., Kahamba, N.F., Ngowo, H.S., & Kaindoa, E.W. (2024) Semi-field evaluation of aquatic predators for the control of *Anopheles funestus* in rural south-eastern Tanzania. Malaria Journal, 23, 228. https://doi.org/10.1186/s12936-024-05055-1
   - Roitberg, B.D., Mondor, E.B., & Tyerman, J.G.A. (2003) Pouncing spider, flying mosquito: blood acquisition increases predation risk in mosquitoes. Behavioral Ecology, 14(5), 736-740. https://doi.org/10.1093/beheco/arg055

- Sirima, D., Oueda, A., Ouedraogo, I., Ouedraogo, I., & Guenda, W. (2017) Distribution des Chironomidae (Dipteres) dans deux reservoirs urbains (Ouagadougou, Burkina Faso). Annale de l'Universite Ouaga. https://progres.ujkz.gov.bf/publications/2902/details#
- Somda, N.S.B., Maiga, H., Mamai, W., Bakhoum, T., Wallner, T., Poda, S.B., Yamada, H., & Bouyer, J. (2022). Adult mosquito predation and potential impact on the sterile mosquito technique. *Scientific Reports*, 12, 2561. https://doi.org/10.1038/s41598-022-06565-1

2. These references informed the mock exercise in PRONTI scoring of potential indicator species shown in Figure 2B.

· *Terrestrial vertebrate predators*

o Ancillotto, L., Falanga, A., Agostinetto, G., Tommasi, N., Garonna, A.P., de Benedetta, F., Bernardo, U., Galimberti, A., Conti, P., & Russo, D. (2023). Predator-prey traits and foraging habitat shape the diet of a common insectivorous bat. *Acta Oecologica*, 118, 03890. https://doi.org/10.1016/j.actao.2023.103890.

o Dietrich, M., Wilkinson, D.A., Le Minter, G., Turpin, M., Galan, M., Charbonnel, N., Dupuy, G., Habchi-Hanriot, N., Reynaud, B., Delatte, H., & Lebarbenchon, C. (2025.) Urban bats change the menu: dietary plasticity across human-modified landscapes of a tropical island. *bioRxiv* 2025.05.06.652573. https://doi.org/10.1101/2025.05.06.652573

o Gonsalves, L., Law, B., Webb, C., & Monam,y V. (2013). Foraging Ranges of Insectivorous Bats Shift Relative to Changes in Mosquito Abundance. *PLoS ONE,* 8(5), e64081. https://doi.org/10.1371/journal.pone.0064081

o Jones. G,, Jacobs. D.S,, Kunz, T.H., Willig, M.R., & Racey, P.A. (2009.) Carpe noctem: the importance of bats as bioindicators. *Endangered Species Research*, 8, 93-115.

o Kasso, M., & Balakrishnan, M. (2013). Ecological and economic importance of bats (Order Chiroptera). *ISRN Biodiversity*, Article No. 187415. https://doi.org/10.1155/2013/187415 https://doi.org/10.3354/esr00182

o Zhu, D., Liu, Y., Gong, L., Si, M., Wang, Q., Feng, J., & Jiang, T. (2024). The Consumption and Diversity Variation Responses of Agricultural Pests and Their Dietary Niche Differentiation in Insectivorous Bats. *Animals*, *14*(5), 815. https://doi.org/10.3390/ani14050815

· *Larvae and adults of other mosquitoes*

o Collins, C.M., Bonds, J.A.S., Quinlan, M.M., & Mumford, J.D. (2019). Effects of the removal or reduction in density of the malaria mosquito, s.l., on interacting predators and competitors in local ecosystems. *Medical and Veterinary Entomology*, *33*(1), 1–15. https://doi.org/10.1111/mve.12327

o Hinne, I.A., Attah, S.K., Mensah, B.A. (2021). Larval habitat diversity and *Anopheles* mosquito species distribution in different ecological zones in Ghana. *Parasites Vectors*, 193. https://doi.org/10.1186/s13071-021-04701-w

o Qureshi, A., Connolly, J.B. (2021). A systematic review assessing the potential for release of vector species from competition following insecticide-based population suppression of *Anopheles* species in Africa. *Parasites Vectors,* 14, 462. https://doi.org/10.1186/s13071-021-04975-0

o Schneider, P., Takken, & W.K Mccall, P.J. (2001). Interspecific competition between sibling species larvae of *Anopheles arabiensis and An. gambiae*. *Medical and Veterinary Entomology*, 14, 165-179. https://doi.org/10.1046/j.1365-2915.2000.00204.x

· *Larvae and adults of other aquatic invertebrates*

o Ayieko, M.A., & Oriaro, V. (2008) Consumption, indigenous knowledge and cultural values of the lakefly species within the Lake Victoria region. *African Journal of Environmental Science and Technology*, 2(10). 282-286.

o Derua, Y.A., Kahindi, S.C., Mosha, F.W., Kweka, E.J., Atieli, H.E., Wang, X., Zhou, G., Lee, M.C., Githeko, A.K., & Yan, G. (2018). Microbial larvicides for mosquito control: Impact of long lasting formulations of *Bacillus thuringiensis* var. *israelensis* and *Bacillus sphaericus* on non-target organisms in western Kenya highlands. *Ecology and Evolution*, 8(15), 7563-7573. https://doi.org/10.1002/ece3.4250

o Frouz, J., Matena, J., & Ali, A. (2003). Survival strategies of chironomids (Diptera: Chironomidae) living in temporary habitats: a review. *European Journal of Entomology* 100, 459-465. https://doi.org/10.14411/eje.2003.069

o Ptatscheck, C., Putzki, H., & Traunspurger, W. (2017) Impact of deposit-feeding chironomid larvae (Chironimus riparius) on meiofauna and protozoans. *Freshwater Science*, 36 (4). https://doi.org/10.1086/694461

· *Aquatic invertebrate predators*

o Cuthbert, R.N., Dalu, T., Wasserman, R.j., Weyl, O.L.F., Fronemon, W., Callaghan, A., Coughlin, W.E., & Dick J.T.A. (2020). Alternative prey impedes the efficacy of a natural enemy of mosquitoes. *Biological Control*, 141, 104146. https://doi.org/10.1016/j.biocontrol.2019.104146

o Gimonneau, G., Bouyer, J., Morand, S., Besansky, N.J., Diabate, A., Simard, F.. (2010). A behavioral mechanism underlying ecological divergence in the malaria mosquito Anopheles gambiae. *Behavioral Ecology*, 21(5):1087-1092. https://doi.org/10.1093/beheco/arq114

○ Murdoch, W.W., Scott, M.A., & Ebsworth, P. (1984) Effects of the general predator, Notonecta (Hemiptera) upon a freshwater community. *Journal of Animal Ecology*, 53, 791-808. https://doi.org/10.2307/4660

o Ouedraogo, J., Sawadogo, S.P., Niang, A.. Soulama, A., Yerbanga, S., Ouedraogo, T.F.X., Guissou, C., Dabire, R.K., Muller, R., Gnandine, O., & Diabate, A. (2025). Interaction of predatory

macroinvertebrate communities with malaria vectors in aquatic habitats of three climatic zones in Burkina Faso. *Parasites & Vectors* **18**, 158 (2025). https://doi.org/10.1186/s13071-025-06794-z

○ Toe, I., Kientega, M. Lingani, A.J., Bouyer, J. Traore, N, Karabinta, A., Millogo, A.A., Kabore, J., Diabate, A. & Maiga, H. (2026) Ecological analysis of mosquito larval communities in Burkina Faso to inform environmental monitoring of genetic control programs. *Science Reports* https://doi.org/10.1038/s41598-026-35602-6

· *Aquatic vertebrate predators*

o Atmar, G.L. & Stewart, K.W. (1972). Food, feeding selectivity and ecological efficiencies of *Fundulus notatus* (Cyprinodontidae). *The American Midland Naturalist*, 88(1), 76-89. https://doi.org/10.2307/2424489

o Kumar, R., Muhid, P., Dahms, H.U., Tseng, L.C., & Hwang, J.S. (2008). Potential of three aquatic predators to control mosquitoes in the presence of alternative prey: a comparative experimental assessment. *Marine and Freshwater Research*, 59(9), 817-835. https://doi.org/10.1071/MF07143

o Louca, V., Lucas, M.C., Green, C., Majambere, S., Fillinger, U., & Lindsay, S.W. (2009) Role of Fish as Predators of Mosquito Larvae on the Floodplain of the Gambia River, *Journal of Medical Entomology*, 46(3), 546–556. https://doi.org/10.1603/033.046.0320

· *Aquatic microbes*

o Mutinda, J., Mwanburi, S.M., Oduor, K.O., Omolo, M.V., Ntabo, R.M., Gathiru, J.M. Mwangangi, J., & Nonoh, J.O.M. (2023) Profiles of bacterial communities and environmental factors associated with proliferation of malaria vector mosquitoes within the Kenyan coast. *Access Microbiology*, 5(8), 5:000606.v4. https://doi.org/10.1099/acmi.0.000606.v4

o Ranasinghe, H.A.K., & Amarasinghe, L.D. (2020) Naturally occurring microbiota associated with mosquito breeding habitats and their effects on mosquito larvae. *BioMed Research International*, 2020, Article 4065315. https://doi.org/10.1155/2020/4065315

· Terrestrial invertebrate predators

- Battiston, R., di Pietro, W., Amerini, R., & Sciberras, A. (2020). The praying mantids (Insecta: Mantodea) as indicators for biodiversity and environmental conservation: a case study from the Maltese and Balearic archipelagos. *Biodiversity*, *21*(3), 142–149. https://doi.org/10.1080/14888386.2020.1848623
- Fagan, W.F., Moran, M.D., Rango, J.J., & Hurd, L.E. (2002). Community effects of praying mantids: a meta-analysis of the influences of species identity and experimental design. *Ecological Entomology*, 27(4), 385-395. https://doi.org/10.1046/j.1365-2311.2002.00425.x
- Greyvenstein, B., du Plessis, H., & van den Berg, J. (2020). The charismatic praying mantid: A gateway for insect conservation. *African Zoology*, *55*(1), 109–118. https://doi.org/10.1080/15627020.2020.1732834
- Kuber, P,. & Gabche, R. (2025) Mismatched menu: the incompatibility of adult black soldier flies as praying mantis feed. *Frontiers in Insect Science*, 5. https://doi.org/10.3389/finsc.2025.1531683
- Somda, N.S.B., Maiga, H., Mamai, W., Bakhoum, T., Wallner, T., Poda, S.B., Yamada, H., & Bouyer, J. (2022). Adult mosquito predation and potential impact on the sterile mosquito technique. *Scientific Reports*, 12, 2561. https://doi.org/10.1038/s41598-022-06565-1

· *Invertebrate pollinators*

- Baldwin, R., Pereira, R.M., Koehler, P.G., & Xue, R-D. (2024) Insecticide toxicity to honey bees: lessons learned from studies by the University of Florida Urban Entomology Laboratory. *Journal of the Florida Mosquito Control Association*, 71(1). https://doi.org/10.32473/jfmca.71.1.135290
- Khalifa, S. A. M., Elshafiey, E. H., Shetaia, A. A., El-Wahed, A. A. A., Algethami, A. F., Musharraf, S. G., AlAjmi, M. F., Zhao, C., Masry, S. H. D., Abdel-Daim, M. M., Halabi, M. F., Kai, G., Al Naggar, Y., Bishr, M., Diab, M. A. M., & El-Seedi, H. R. (2021). Overview of Bee Pollination and Its Economic Value for Crop Production. *Insects*, *12*(8), 688. https://doi.org/10.3390/insects12080688

o van Huis, A. (2021) Cultural aspects of ants, bees and wasps, and their products in sub-Saharan Africa. *Int J Trop Insect Sci,* 41, 2223–2235. https://doi.org/10.1007/s42690-020-00410-6

o Robert, O.S., Labrie, G., Chagnon, M. & Fournier, V. (2014). Neonicotinoid-contaminated puddles of water represent a risk of intoxication for honey bees. PLOS One 9(12): e108443. https://doi.org/10.1371/journal.pone.0108443
